# An experimental investigation into scope rigidity in written Mandarin

**DOI:** 10.3389/fpsyg.2023.1128616

**Published:** 2023-06-09

**Authors:** Hongchen Wu

**Affiliations:** School of Modern Languages, Georgia Institute of Technology, Atlanta, GA, United States

**Keywords:** inverse scope, adverbial clause, speaker variation, verb type, numbers, truth-value judgment task

## Abstract

Mandarin Chinese is claimed to be a scope-rigid language, as its doubly-quantified simple transitive sentences are unambiguous with surface scope only and no inverse scope available. However, it has been debated whether Mandarin Chinese allows inverse scope in some syntactic environments other than simple transitives. This paper investigates whether scope rigidity as a property of the grammar of Mandarin prevents scope ambiguity in different syntactic environments and what factors influence scope interpretations. Using a Truth-Value Judgment task, we tested the judgments of 98 Mandarin Chinese native speakers on transitive sentences containing both a subject and object quantifier under adverbial clauses. The results show that inverse scope reading is considered available for doubly-quantified transitives under adverbial clauses, although there are intra-participant variances. The results challenge the well-established approaches to quantifier scope in Mandarin and call for rethinking the long-standing dichotomy view of quantifier scope in languages. We also found bimodal distribution on the acceptance of inverse scope readings, suggesting that there may be two different populations of native speakers with two different grammars. In addition, we also observed other factors that may affect scope behaviors, including clause type, presence of aspect marker, verb type, and numbers.

## 1. Introduction

As in (1) and (2), English and Mandarin both allow surface scope readings, i.e., the existential quantifier scopes over the universal quantifier, just as the linear order shows.^1^ What distinguishes English from Mandarin is the availability of the inverse scope reading in a simple transitive sentence. In English, the universal quantifier *every* can scope over the existential quantifier *a* when *every* is linearly preceded by *a*. However, such inverse-scope reading is generally argued to be unavailable in Mandarin.

(1)  A girl read every book.
     a. a > every: ‘There is a particular girl *x* such that *x* read every book.’
     b. every > a: ‘For every book *y*, a (possibly different) girl read it.’

(2)  *(Yǒu-)yī-gè-nǚhái*     *dú-le*     *měi-běn-shū.*
     (have-)one-clf-girl     read-asp     every-clf-book
     ‘A girl read every book.’
     a. a > every: ‘There is a particular girl x such that x read every book.’
     b. *every > a: ‘For every book y, a (possibly different) girl read it.’

As a canonical construction in a language, the scope behavior of simple transitives is naturally considered to represent quantifier scope. Thus, English is argued to be a scope-fluid language that allows both surface scope reading and inverse scope reading, while Mandarin is claimed to be a scope-rigid language that only allows surface scope (Huang, 1981, 1982; Lee, 1986; Aoun and Li, 1989, 1993, among others). Scope rigidity as a feature of Mandarin has gradually become well-known. Mandarin and languages with Mandarin-like scope behaviors (such as Japanese, see Hoji, 1985), are often referred to as languages lacking scope ambiguity or obeying scope isomorphism in the linguistic field since the 1980s.

However, as noticed over the years, scope ambiguity cases in Mandarin are not unusual and have been observed in PP datives, PP locative, non-finite clauses, relative clauses, and thetic sentences, resembling their English counterparts concerning scope interpretation, although many of the observed scope ambiguity is not experimentally confirmed yet (Chien and Wexler, 1989; Lee, 1989, 1991, 2002; Chien, 1994; Lee et al., 1999; Lin, 2013, 2015; Liu and Wu, 2016; Wu et al., 2018; Chen, 2020; Gan, 2021, among others). For example, (3).

(3)  a. *Lǎo shī*     *sòng-le*     *(yì)xiē-píngyǔ*     *gěi*     *měi-gè-xuésheng.*
     teacher give-asp some-comment to every-clf-student
     ∃ > ∀: ‘some comment x is such that the teacher gave x to every student.’
     ∀ > ∃: ‘every student y is such y was given a (possibly different) comment by the teacher.’ (Liu and Wu, 2016)
     b. The teacher gave some comment to every student. (∃ > ∀, ∀ > ∃)

But the literature debates over whether simple transitives under a non-matrix clause environment show scope ambiguity in Mandarin. For instance, (4).

(4)  *Yàoshì*     *liǎng-gè-rén*     *zhǎodào*     *měi-gè-xiànsuǒ*
     if          two-clf-men     found     every-clf-clue
     2 > ∀: ‘two persons *x* are such that *x* found every clue.’
     *∀ > 2: ‘every clue *x* is such that two persons found *x*.’ (Aoun and Li, 1989, ex. 1b)

Aoun and Li (1989) argue that (4) is not ambiguous, like the matrix simple transitives in (2) Mandarin. However, more recent studies (Scontras et al., 2016; Grano, 2017) have suggested that conditional clauses may allow scope ambiguity for doubly quantified transitives as conditional clauses are truncated clauses, different from matrix clauses (Haegeman, 2006, 2012).

In the present study, we conducted an untimed, offline experiment using the Truth-Value Judgment task to test if sentences like (4) allow inverse scope reading and investigate how scope interpretations are affected by other factors. We will report findings from the experiment and provide discussions on the scope-rigidity tag on Mandarin and the rigidity-fluidity dichotomy for quantifier scope interpretations.

## 2. Materials and methods

### 2.1. Experiment design

To collect the scope behavior data, we used a Truth-Value Judgment task, which is developed by Crain and McKee (1985) and Crain and Thornton (1998) and has been frequently used in experimental studies on scope interpretation, such as Su (2001), Su and Crain (2013), Scontras et al. (2016, 2017), among others.

Previous studies using truth-value judgment tasks have employed different ways to present the given context that leads to a certain scope reading. A traditional way is to use pictures to present the context, as used by Scontras et al., 2016, 2017. An alternative way is to use narrative storytelling to describe a context that leads to a specific scope interpretation, such as, acting out detailed storytelling used in Su (2001), and short written narratives used by Zhou and Gao (2009). In the present study, we need to illustrate the relation between adverbial clauses and the main clauses, a relation that is not easily presented in a single picture. But, written narrative storytelling has the advantage of describing complex contexts thoroughly and vividly. Therefore, we used written stories to set up the contexts instead of pictures. With the intention of engaging participants in the scope judgment task, we followed the detailed storytelling style used by Su (2001) and created a 150-word written narrative for each context in this study. Each narrative describes a specific and real-life scenario which includes clear background information and gives the specific name(s) to the characters(s) involved in the story. For example, a narrative starts with a stadium holding public events with insufficient security in the past, then talks about the security manager Mr. Li holding a security meeting to make new arrangements for doing security checks at the gateway, and finally ends with the proposed changes on the security check.

An example of a written story is in (5), which is a context to force the inverse scope reading of the target sentence in (6).^2^

(5)  以往新华体育馆办大型活动时，每个出口只有一名警察看守。由于安保力量不够，以前发生过多次小偷偷窃钱包、最后小偷逃走的事情。最近的新华体育馆安保讨论会上，大家跟安保负责人老李建议:加强安保力量，每个出口都安排三名警察把守，检查出入人员。这样的话，即使发生小偷偷窃东西的事情，小偷也不可能从活动现场溜走。
       *[English translation of the context]:*

‘In the past, when Xinhua Stadium held a public event, there was only one police guard at each exit. Due to insufficient security, there have been many thieves stealing wallets and fleeing at the end. At a recent meeting about security, the staff suggested to Mr. Li, the security manager, that the security forces should be strengthened by putting three police guards at each exit and policemen can do security check at the gateway. This way, even if a thief stole something, it is unlikely that the thief could easily slip away from the Stadium.’

(6)  要是三名警察看守每个出口，小偷就不可能从活动现场溜走。
     *[word-for-word glosses of the target sentence]:*
     *Yàoshì*     *sān-míng-jǐngchá*     *kānshǒu*     *měi-gè-chūkǒu*,
     if     three-clf-policeman     guard     every-clf-exit
     *xiǎotōu*     *jiù*     *bù*     *kěnéng*     *cóng*
     thief then not possible from
     *huódòng-xiàncháng*     *liūzǒu.*
     event.site slip.away
     ‘If three police officers guard each exit, a thief is unlikely to slip away from the Stadium.’

Considering that it has been claimed that Mandarin adverbial clauses show similar central-peripheral distinctions with respect to structural properties as English adverbial clauses do (Haegeman, 2002 and following work about English, Lu, 2003, 2008, and Wei, 2018, and Wei and Li, 2018 on Mandarin) and clause size is suspected to be relevant to scope ambiguity (Grano, 2017; Wu et al., 2018), adverbial clause types are varied across conditions for the stimuli design. In addition to conditional clauses (*yàoshì* ‘if’), the stimuli also include concessive clauses (*suīrán* ‘although’) and reason clauses (*yīnwèi ‘*because*’*) to test whether adverbial clause type may affect scope interpretation. The presence of the aspectual marker *le* is considered as another factor for the stimuli since finiteness has been argued to be relevant to the clause size and scope interpretation (see Lin, 2013, 2015; Grano, 2017 in particular). Therefore, two factors were controlled for the stimuli: the type of adverbial clauses (*yàoshì* ‘if’, *suīrán* ‘although’, *yīnwèi* ‘because’) and the presence of the aspectual marker *le*; there are 6 target conditions in this study.

All adverbial clauses in the stimuli are positioned sentence-initially with the order of “adverbial clause - main clause,” as the sentence-initial position is the preferred position for most adverbial clauses while sentence-final adverbial clauses in Mandarin are generally considered as marked or less preferred or “unplanned” utterances (Chao, 1968: 132–133). Moreover, paired conjunctions (e.g., *yīnwèi…, suǒyǐ…* ‘because…, so…’) are not used, since the two clauses with paired conjunctions are argued to be root clauses with a coordinated structure (Wei, 2018).^3^

Inside the adverbial clauses, the existential quantificational phrase linearly precedes the universal quantificational phrase: one in the embedded subject position, the other in the embedded object position. The universal quantificational phrases are all in the form of *mei* ‘every’ + classifier + noun, while for the existential quantificational phrases, the form of *yī* (‘a/one’) */ liǎng* (‘two’)*/sān* (‘three’) + classifier + noun is used, and the occurrence of *yī*, *liǎng* and *sān* (‘three’) are balanced for the stimuli. Varying the existential quantificational phrases is to incorporate the *a* versus *one* debate on Mandarin *yī* (‘a/one’) and to avoid the potential influence of participants interpreting Mandarin *yi* as a single referent rather than an indefinite (Lee, 1986; Chien, 1994; Liu, 1997; Scontras et al., 2017; Yang and Wu, 2020).

In addition, considering that the lexical information of verbs may have effects on scope judgments (Zhou and Gao, 2009) and the aspect marker *le* tends to indicate the completion of an event and combine with resultative verbs, we balanced two different types of verbs for the stimuli as well: resultative verbs (*chīdiào* ‘eat up’, *dáduì* ‘answer (questions) correctly’, *dāchū* ‘build up’)^4^, and durative verbs (*xiézhù* ‘assist’, *bùzhì* ‘decorate (a room)’, *kānshǒu* ‘guard’). Compared with durative verbs, resultative verbs have a natural ending point of an event and are more naturally compatible with *le* (an event realization operator, see Jo-wang Lin, 2003a for more discussion on *le*).

Table 1 presents the stimuli paradigm. 6 sets of 6 sentences with one target sentence for each condition in each set were created as the target sentences. QNP represents quantificational phrases in Table 1 and henceforth.

**Table 1:** The stimuli paradigm.

Condition	Structure of the target adverbial clauses	Type of adverbial clauses	Presence of *le* inside adverbial clauses
*suīrán_le*	*Suīrán* QNP Verb-*le* QNP, …	*suīrán* ‘although’	YES
*yīnwèi_le*	*Yīnwèi* QNP Verb-*le* QNP, …	*yīnwèi* ‘because’	YES
*yàoshì_le*	*yàoshì* QNP Verb-*le* QNP, …	*yàoshì* ‘if’	YES
*suīrán_no le*	*Suīrán* QNP Verb QNP, …	*suīrán* ‘although’	NO
*yīnwèi_no le*	*Yīnwèi* QNP Verb QNP, …	*yīnwèi* ‘because’	NO
*yàoshì_no le*	*Yàoshì* QNP Verb-le QNP, …	*yàoshì* ‘if’	NO

Since the previous studies diverge from each other on the availability of inverse scope reading in conditional clauses, we focused on surveying the availability of inverse scope in the present study. For each target sentence, only the corresponding context that leads to the inverse scope reading of that sentence is provided. 36 target sentences were randomized with 108 fillers and distributed across 6 lists in a Latin Square Design. Each participant was presented with 6 target sentences (one sentence for each condition) intermingled with 18 fillers. A full list of the target sentences is provided in the Supplementary materials.^5^

### 2.2. Procedures

**Figure 1:**
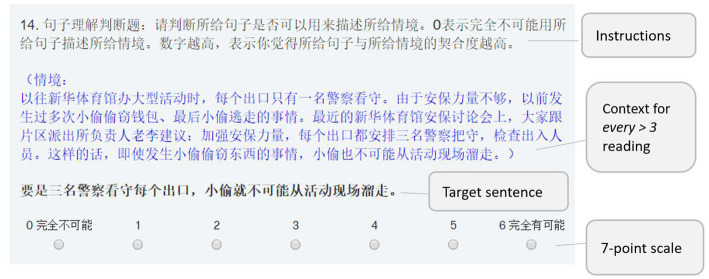
The display sample of the online survey.

The untimed experiment was conducted through the online survey platform Qualtrics. No time limitation on completion was enforced. Participants were given a practice session to get familiar with the format of the judgment task. All sentences including instructions were fully displayed on the screen with simplified Chinese characters. Figure 1 is a screenshot of the online survey. Participants were asked to rate on a 7-point scale (0: completely impossible; 6: perfectly possible) to judge whether the target sentence is possible to be used to describe the given contexts. They needed to click the button representing the numerical rating to indicate their judgment.

### 2.3. Participants

Participants were recruited through social media and emails. Participation in this experiment was completely anonymous. 168 native Mandarin speakers participated in this experiment. 98 of them completed the survey and only their data were included in the analysis and results reported below. Among the 98 participants, the number of female participants was 68 and the average age of these 98 participants was 29.7 (the age range was 19–55).

## 3. Results

Data were processed in the R software environment (version: 3.5.3, R Development Core Team, 2019). To check the statistical significance, the *lme4* package (version 1.1-21, developed by Bates et al., 2015a) was used to perform a linear mixed-effects model.^6^

In the literature that used a linear mixed-effects model to analyze acceptability rating, studies differed in their choices of random-effects structures: some studies used a random intercept model and take by-subject variation into consideration (Brendel, 2019), while other studies used a more complex random-effect structure – a random slope model and modeled by-subject and by-item variability in how conditions affect acceptability ratings Harris et al. (2013). In the present study, we used a linear mixed-effects model with two fixed factors “Type of adverbial clauses” and “The presence of *le* inside adverbial clauses” (as indicated in the experiment design table, i.e., Table 1), and two random intercept effects “participant” and “set” for different participants and a different set of stimuli.

The random intercept model was chosen instead of a random slope model for two reasons. First, although we expect general baseline by-subject and by-item variability in the acceptability ratings, we do not have clear empirical reasons to assume that the effect of fixed factors, such as the presence of *le*, might be different for different subjects. The two fixed factors we controlled here are *presence of le, and adverbial type.* They are different from fixed factors like *politeness.* For *politeness,* we would use a random slope model and assume the effect of politeness can be different for different subjects, since some subjects may have higher standards for politeness than others. Second, our data do not support a complex random effect structure with random slopes. When fitting a random slope model, a warning massage from R is returned suggesting that this model has a singular fit. After checking singularity for this model using isSignular() function, we used rePCA() to perform a Principal Components Analysis (PCA) for this random slope model and the PCA result shows that such a random slope model can be overparameterized (Bates et al., 2015b; Bross, 2019). In contrast, the random intercept model used in the present study passed both the singularity check and PCA check. ^7^

**Figure 2:**
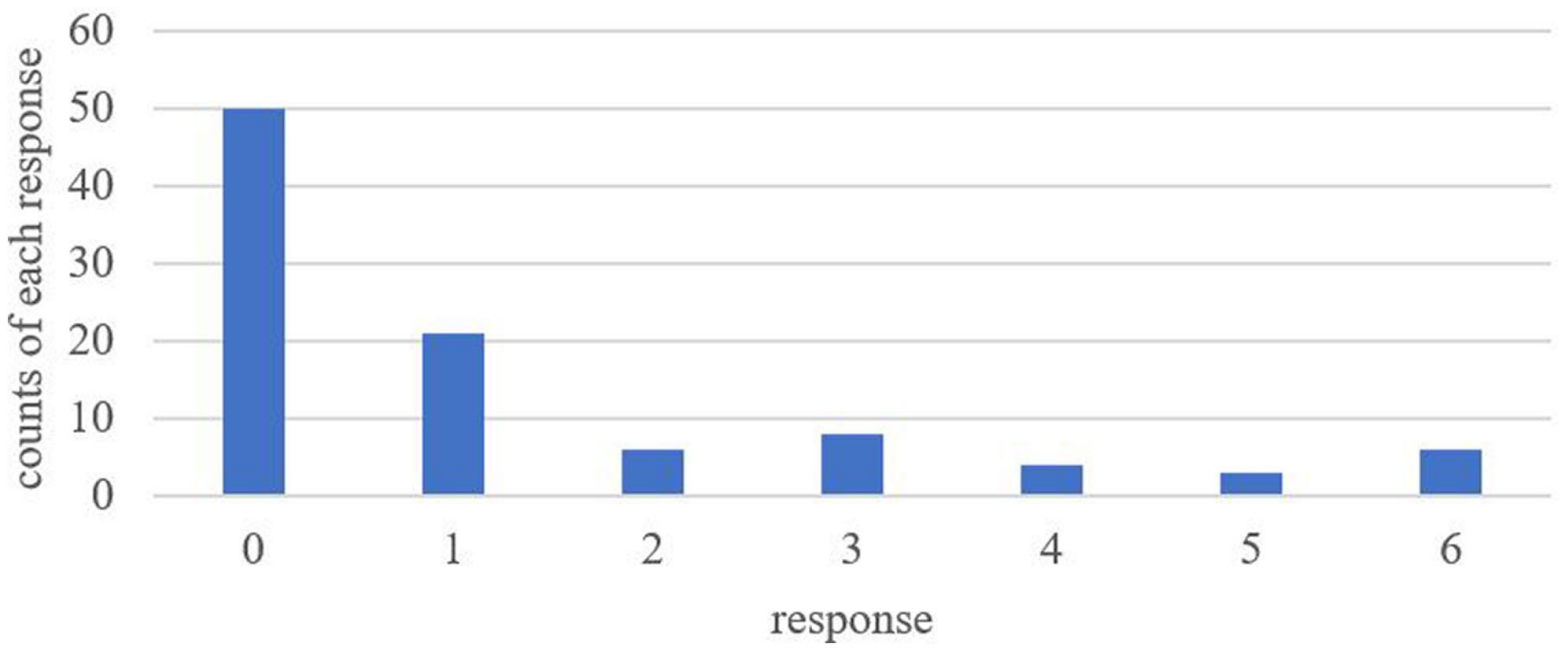
Distribution of ratings of context-sentence compatibility on simple actives (*N* = 98).

Before presenting the results of the target sentences in 6 conditions, we will present results from the fillers as the baseline. Among the fillers included in this experiment, there are simple actives like (7) and the given context for these sentences leads to an inverse scope reading as well.^8^ The mean acceptance rate of such context-sentence pairs is 1.27 and the corresponding distribution of the acceptance rates is shown in Figure 2.

(7)  *Yī-gè-nánhái*     *tiàoguò-le*     *měi-gè-lángān.*
     one-clf-boy     jump.over-pfv     every-clf-fence
     ‘A boy jumped over every fence.’
     (*Context given along with this sentence*: The team of Xiao Wang, Xiao Li, and Xiao Ming was shortlisted for the Men’s High Jump Team Finals. There are three crossbars of different heights. Xiao Wang jumped over the first crossbar, then Xiao Li jumped over the second one, and Xiao Ming jumped over the third.)

Figure 2 shows that the matching between an inverse scope context and a simple active sentence like (7) was rated with a very low score. More than 75% of the participants (77 out of 98 participants) rated below 3, which indicates that most of the participants did not consider the simple actives able to be used to describe an inverse scope context. This result echoes previous studies and claims on the scope rigidity of doubly-quantified simple actives in Mandarin (Su, 2001; Scontras et al., 2016; Gan, 2021).

PP datives like (8) and its minimal pairs Double-Object Constructions (DOCs) like (9) were included as fillers as well.^9^ The mean acceptance rate of context-sentence pairs like (8) is 3.33 while the mean acceptance rate of context-sentence pairs like (9) is 1.59.

(8)  *Xiǎolì*     *sòng-le*     *yī-jiàn-lǐwù*     *gěi*     *měi-gè-mèimei.*
     Xiaoli gave-asp     one-clf-gift to     every-clf-younger.sister
     ‘Xiaoli gave a gift to every younger sister.’
     (*Context given along with this sentence:* When going back home to celebrate Spring Festival, Xiaoli bought each of her younger sisters a gift.)

(9)  *Xiǎolì*     *sòng-le*     *yī-ge-mèimei*     *měi-jiàn-lǐwù.*
     Xiaoli gave-asp     one-clf-younger.sister     every-clf-gift
     ‘Xiaoli gave a younger sister every gift.’
     (*Context given along with this sentence:* When going back to her hometown for celebrating Spring Festival, Xiaoli bought each of her younger sisters a gift.)

**Figure 3:**
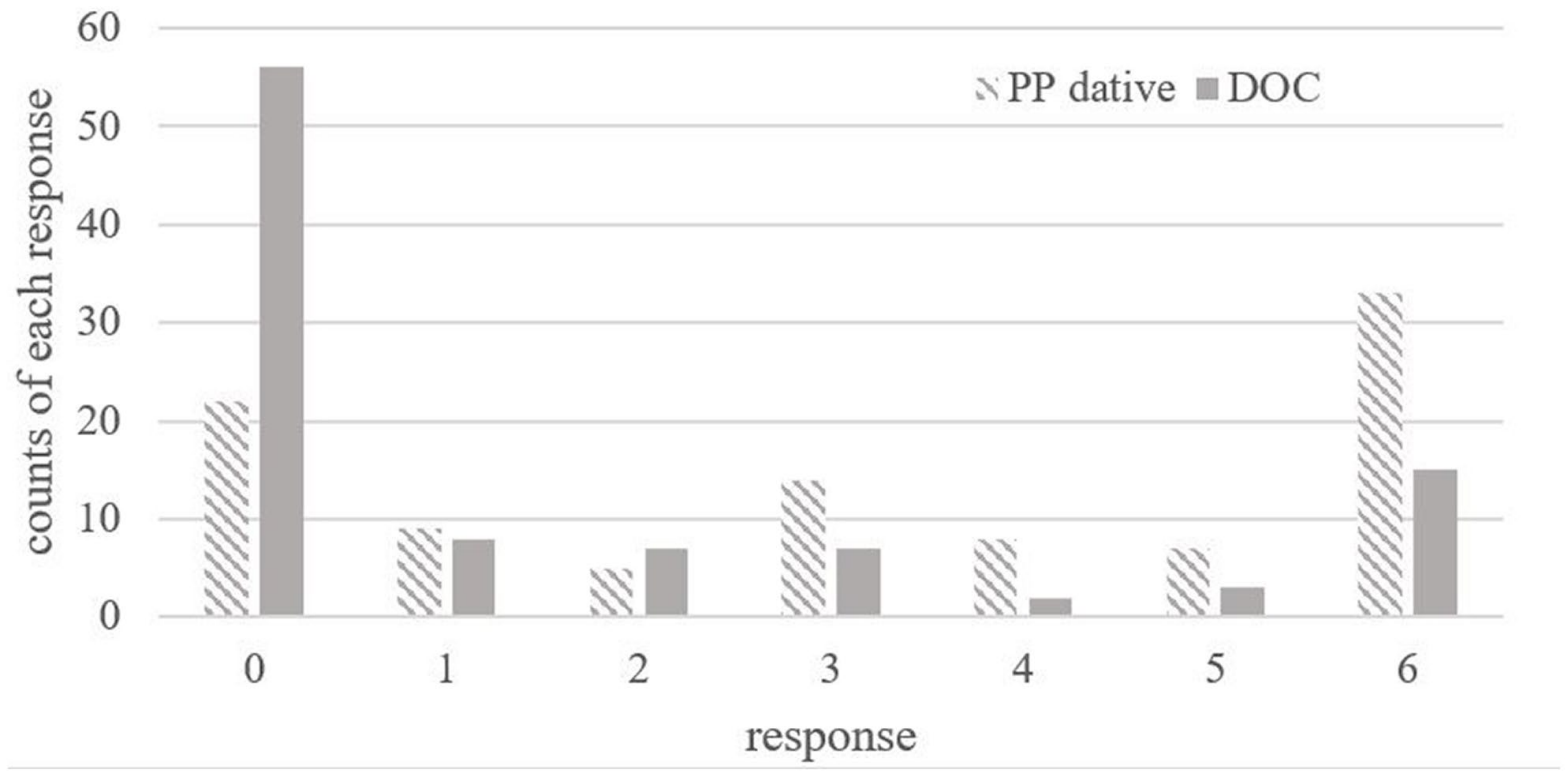
Distribution of ratings of context-sentence compatibility on PP datives and Doble Object Constructions (DOC) (*N* = 98).

Figure 3 shows the distribution of the acceptance rates of PP datives and DOCs: the former was rated with a score greater than or equal to 3 by 63.27% of participants (62 out of 98 participants) but the latter was rated with a score below 3 by 72.44% of the participants (71 out of 98 participants). This suggests that most participants think that PP datives can be used to describe an inverse scope context much more freely than DOCs. This result is in line with findings from studies like Su (2001:53), Gan (2021) and theoretical claims made in studies like Huang (1982), and Aoun and Li (1989: 167).

One may wonder why the mean rating of the context-sentence compatibility on PP datives (a claimed-to-be ambiguous case) is only 3.33, not very close to the possibly highest score 6. As noticed in previous studies, inverse interpretations come at a cost (Anderson, 2004, chapter 2, among others). For example, Scontras et al. (2017) report that the mean ratings for inverse scope reading for English doubly quantified transitives (similar to the (1)) were 4.46 (out of 7), a similar result as the mean ratings for the inverse scope in Mandarin PP datives (3.33 out of 6 in the present study). One may also wonder why the mean rating of context-sentence compatibility on DOCs (a claimed-to-be unambiguous case) is not at or near the floor. As a matter of fact, similar ratings were reported in previous studies, like Gan (2021): the acceptance rate for the picture representing inverse scope reading of sentence like (9) is 16.67%, not that close to 0% either. Meanwhile, experimental studies on English DOCs (also a claimed-to-be unambiguous case) have shown that inverse scope reading although not preferred but available for speakers. For example, Heizmann (2007) reports that inverse scope is accessible for participants 40% of the time for the double object constructions like “*Christine showed a visitor every picture by Picasso.*.” These experimental results suggest that double object constructions in both Mandarin and English may allow some availability of inverse scope reading for some speakers. The speaker variations found in these studies lead us to rethink the dichotomy of scope interpretations, which we will discuss in detail in Section 4.

**Figure 4:**
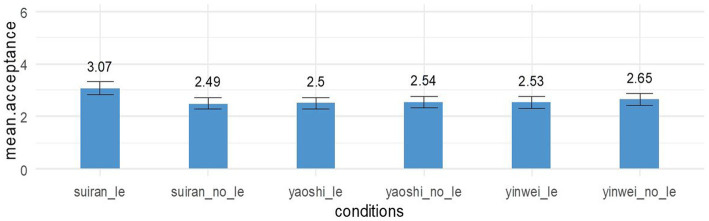
Mean ratings of context-sentence compatibility in the target conditions (*N* = 98) (Note: *suīrán* ‘although’, *yàoshì* ‘if’, *yīnwèi* ‘because’; le is an aspectual marker).

The mean acceptance rates of the 6 target conditions are shown in Figure 4. The mean ratings of context-sentence matching pairs in each condition were generally around 2.5, significantly higher than the mean ratings for the claimed-to-be unambiguous DOCs and simple actives, but lower than the mean ratings for the ambiguous PP datives.

Before running the comparison between any two conditions using the formula provided earlier^10^, we used anova() function to compare an inter-dependent model (response ~ adverbial_type * presence_le) and a non- inter-dependent model (response ~ adverbial_type + presence_le) to check whether these two factors are inter-dependent on each other. The results returned by the anova() show that no significant interaction effect was found between adverbial types and presence of *le* [β_yàoshì:presence_le_ = −0.61671, SE = 0.37963, β*_yīnwèi:presence_le_* − 0.69506, SE = 0.37960, χ^2^(2) = 4.0064, *p* = 0.1349].

Now let us look at the statistical results from the comparison of every two conditions. The formula for the full model is either *fm.full1 < − lmer(response ~ adverbial_type + (1|participant) + (1|set), data = data, REML = FALSE)*, or *fm.full2 < − lmer(response ~ presence_le + (1|participant) + (1|set), data = data, REML = FALSE)*, depending on which two conditions were compared. The reduced model is *fm.reduced < − lmer(response* ~ *(1|participant) + (1|set), data = data, REML = FALSE)*. For example, the fm.full2 model was compared with the fm.reduced model when performing statistical comparisons between the *suīrán_le* condition and the *suīrán_no le* condition, since the two conditions only differ in the presence of *le*. In contrast, when comparing two conditions which only differ in the adverbial clause type, such as the *suīrán_le* condition and *yàoshì_le* condition, the fm.full1 model was compared with the fm.reduced2 model. The test for checking statistical significance among conditions was performed though the likelihood ratio test using the anova() function (Winter, 2013). The *value of p* returned by *anova (fm.full, fm.reduced)* represents the effect of the factor “Type of adverbial clauses” or “The presence of *le* inside adverbial clauses” on the difference between the acceptability rates (i.e., “Response”) of two conditions.

To check the effect of adverbial types, we used *anova (fm.full1, fm.reduced)*. When *le* is not present, no significant differences in the ratings were found among the three conditions: *suīrán_no le* condition (mean = 2.49, 95% confidence intervals of the mean = 2.05–2.93, standard error = 0.22), the *yàoshì_no le* condition (mean = 2.49, 95% confidence intervals of the mean = 2.05–2.93, standard error = 0.22), and *yīnwèi_no le* condition (mean = 2.65, 95% confidence intervals of the mean = 2.18–3.13, standard error = 0.24). *Suīrán_no_le* condition was not rated significantly higher than *yīnwèi_no_le* condition [β = 0.1944, SE = 0.2597, χ^2^(1) = 0.5529, *p* = 0.4571], and *yàoshì_no_le* condition [β = 0.05527, SE = 0.27479, χ^2^(1) = 0.0401, *p* = 0.8413]. The ratings for *yīnwèi_no_le* condition were not significantly different from the ratings for *yàoshì_no_le* either [β = 0.05527, SE = 0.27479, χ^2^(1) = 0.0401, p = 0.8413].

Among the three conditions where *le* is present, adverbial type significantly affected ratings: context-sentence ratings on *suīrán_le* condition (mean = 3.07, 95% confidence intervals of the mean = 2.56–3.58, standard error = 0.25) were significantly higher than ratings on *yīnwèi*_*le* target sentences [mean = 2.53, 95% confidence intervals of the mean = 2.05–3.01, standard error = 0.24, β = −0.5373, SE = 0.2682, χ^2^(1) = 3.9649, *p* = 0.04646], and on *yàoshì_le* target sentences (mean = 2.53, 95% confidence intervals of the mean = 2.05–3.01, standard error = 0.24, β = −0.5708, SE = 0.2745, χ^2^(1) = 4.2607, *p* = 0.039). However, no significant difference in the ratings was found between the *yīnwèi_le* condition and the *yàoshì_le* condition [β = 0.008792, SE = 0.276280, χ^2^(1) = 0.0018, *p* = 0.966].

The comparison formula for checking the effect of presence of *le* is: anova (fm.full2, fm.reduced), where the two models differ only in whether *presence_le* is included as a fixed factor. There was no significant difference between the *yàoshì_le* condition and the *yàoshì_no le* condition [β=0.04082, SE = 0.29403, χ^2^(1) = 0.0195, *p* = 0.889], nor between the *yīnwèi_le* condition and the *yīnwèi_no le* condition [β = −0.1107, SE = 0.2666, χ^2^(1) = 0.1764, *p* = 0.6745]. However, *suīrán_le* was rated significantly higher than its minimal pair condition *suīrán_no le* [β = 0.5913, SE = 0.2484, χ^2^(1) = 5.526, *p* = 0.01874]. In a word, the *suīrán_le* condition is the only condition among the six conditions that show a significant difference from its minimal pair conditions.

The results also show that for the conditions with the presence of *le*, the mean ratings on context-sentence compatibility of target adverbial clauses with resultative verbs are higher than the target adverbial clauses with durative verbs, while the pattern is reversed for conditions without the presence of *le* (Table 2). To check if the inter-dependence exists between verb types and presence of *le,* we used anova(fm.verb1, fm.verb2) where fm.verb1 < − *lmer*(response ~ adverbial_type + presence_le * VerbType + (1|participant) + (1|set), data = data, REML = FALSE) and fm.verb2 < − *lmer(response ~ adverbial_type + presence_le + VerbType +  (1| participant) + (1 | set), data = data,REML = FALSE)*. The interaction between the presence of *le* and the verb type was found to be significant: context-sentence ratings on target sentences with durative verbs and *le* were significantly different from the ratings on other target sentences [β = −1.0629, SE = 0.3613, χ^2^(1) = 8.5726, *p* = 0.003413].

**Table 2:** The mean ratings of context-sentence compatibility of the target conditions by the types of verbs inside the adverbial clauses (*N* = 98).

Condition	Resultative verbs	Durative verbs
*suīrán_le*	3.2	2.94
*suīrán_*no *le*	2.22	2.76
*yàoshì_le*	2.71	2.28
*yàoshì_*no *le*	2.02	3.02
*yīnwèi_le*	2.58	2.48
*yīnwèi_*no *le*	1.85	3.42

In the experiment design, three different existential quantificational phrases (in the form of number + classifier + noun and positioned in the subject of an adverbial clause) were used across the 6 sets in the stimuli to balance out the potential effect of different numerals on scope interpretation. Table 3 presents the differences in mean ratings caused by differences in existential quantificational phrases: for each target condition, the mean rating is generally higher when the existential quantificational phrases are in the form of *liǎng* (‘two’)*/sān* (‘three’) + classifier + noun than when they are in the form of *yī* (‘a/one’) + classifier + noun.

**Table 3:** The mean ratings of context-sentence compatibility of the target conditions by the types of existential quantificational phrases (*N* = 98).

Condition	Existential QNP_*yī* ‘a/one’	Existential QNP_*liang* ‘two’	Existential QNP_*san* ‘three’
*suīrán_le*	2.82	3.3	3.03
*suīrán_*no *le*	2.07	3.16	2.09
*yàoshì_le*	2.38	2.82	2.29
*yàoshì_*no *le*	2.59	2.67	2.32
*yīnwèi_le*	1.58	2.58	3.35
*yīnwèi_*no *le*	2.15	2.71	3.05

**Figure 5:**
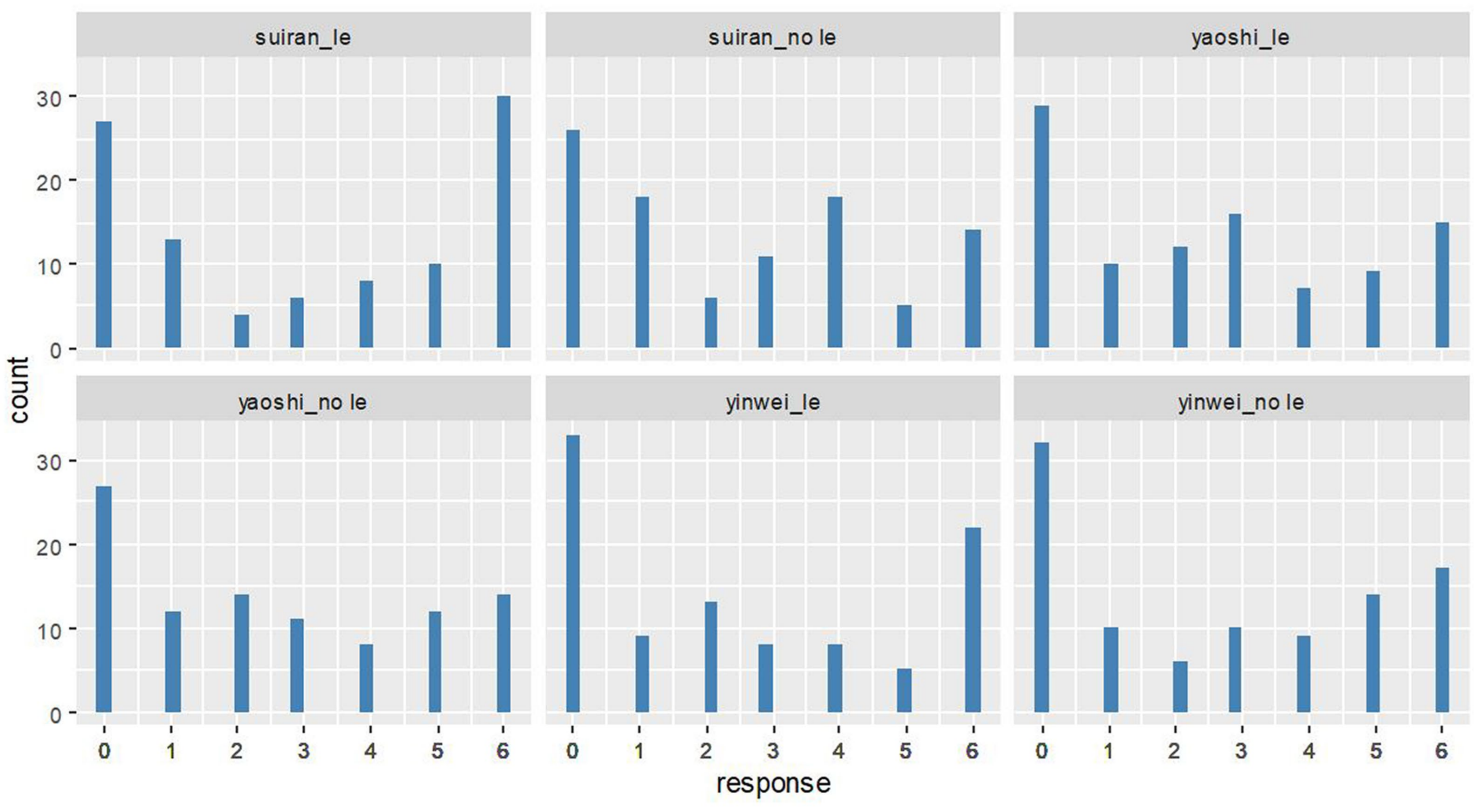
The distribution of ratings on the target conditions (*N* = 98).

**Figure 6:**
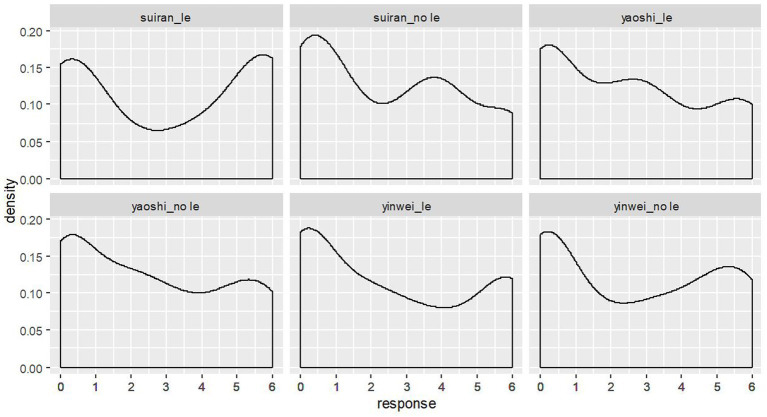
The density plot of ratings on the target conditions (*N* = 98).

In addition, we investigated both intra-participant and by-participant distribution of the ratings to see if and how participants respond differently. Interestingly, the histogram plot in Figure 5 and the density plot in Figure 6 both show a bimodal distribution of the ratings on target context-sentence pairs for each target condition. In other words, about half of the participants think that the target adverbial clauses with the linear order of ∃ over ∀ match a ∀ over ∃ scenario, while the other half of the participants do not think so. Among 98 participants, the number of participants who rated the context-sentence pairs in each condition with a score greater than 2 is as follows:

a) 54 participants (about 55% of all participants) for the condition *suīrán_le*
b) 48 participants (about 49% of all participants) for the condition *suīrán_no le*
c) 47 participants (about 48% of all participants) for the condition *yàoshì_le*
d) 45 participants (about 46% of all participants) for the condition *yàoshì_no le*
e) 43 participants (about 44% of all participants) for the condition *yīnwèi_le*
f) 50 participants (about 51% of all participants) for the condition *yīnwèi_no le*

**Figure 7:**
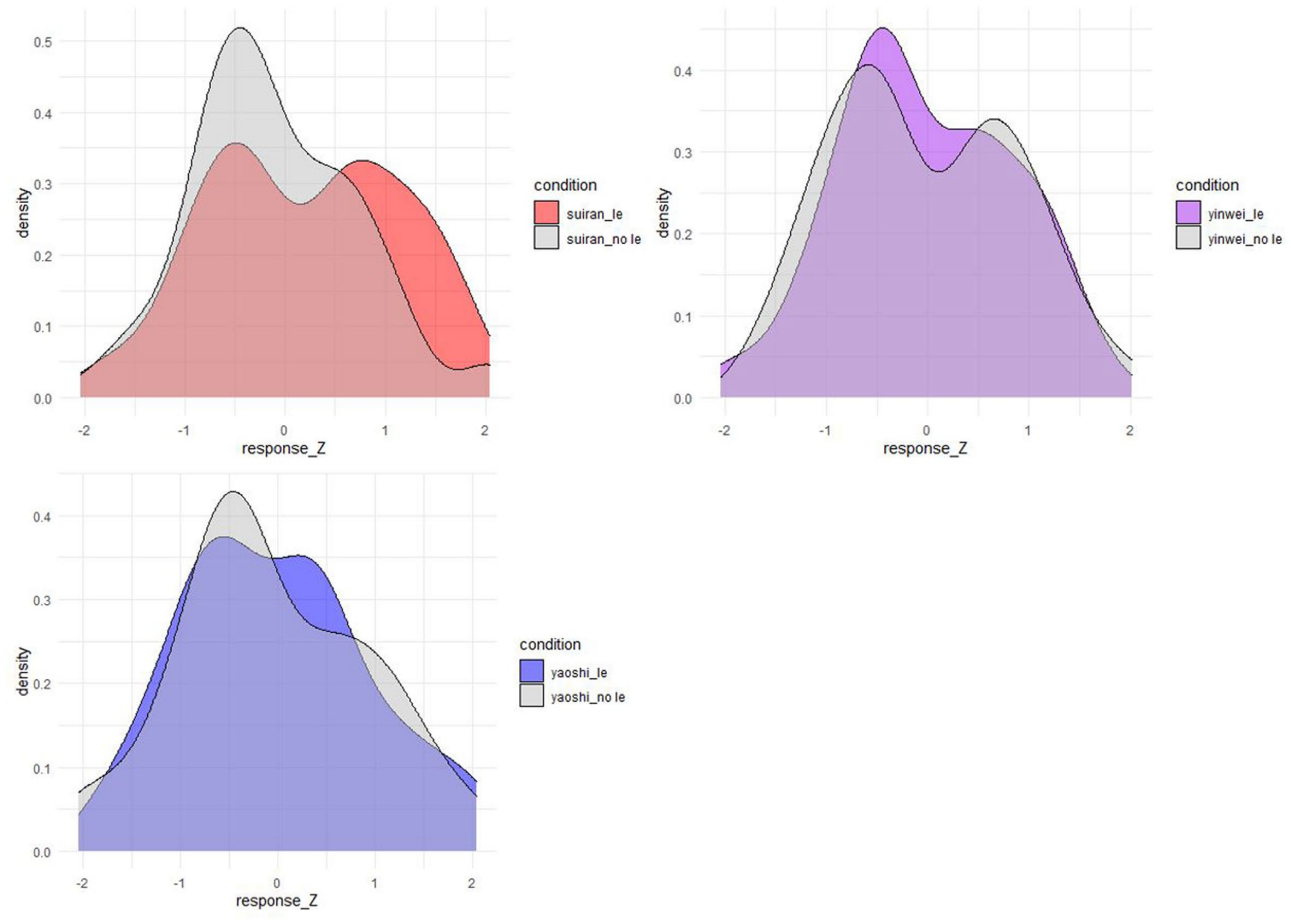
The distribution of *z*-normalized ratings on the target conditions (*N* = 98).

The bimodal distribution of the ratings on target context-sentence pairs is also found after data normalization. We normalized the responses with a z-score and the distribution of normalized ratings is in Figure 7, wherein we can see similar bimodal distribution patterns as we have seen in Figures 5, 6. ^11^

Overall, the results reveal interesting results about the behaviors of quantifier scope in Mandarin adverbial clauses: (a) inverse scope is allowed for Mandarin adverbial clauses although there is variation among participants; (b) the type of adverbial clauses only affects the context-sentence compatibility when *le* is present (the mean ratings of *suīrán_le* is much higher than *yàoshì_le* and *yīnwèi_le*); (c) different verb types are inter-dependent with the presence of *le* factor and together affect context-sentence compatibility; (d) participants tend to give a higher rating on the context-sentence compatibility when the existential quantificational phrases are in the form of *liǎng* (‘two’)*/sān* (‘three’) + classifier + noun compared to *yi* (‘a’/‘one’) + classifier + noun.

## 4. Discussions

Although the mean rating of context-sentence matching pairs in each target condition (Figure 4) is just in the range of 2.5–3.0, the results still confirm that it is possible to have inverse scope reading of doubly-quantified adverbial clauses. Scontras et al. (2017) reported an average rating of 4.46 on a 1 to 7 scale for inverse scope reading and claimed the availability of inverse scope reading in English simple transitives based on this rating. Our results are based on a 0 to 6 scale, and the structures examined in the present paper are simple transitives under adverbial clauses, a much more complex structure than the ones examined in Scontras et al. (2017). Complex structures generally are associated with lower ratings (Gibson and Thomas, 1999), so it is expected to see relatively lower mean ratings in this study. Moreover, the mean rating of context-sentence matching pairs in each condition is much higher than the mean rating of doubly-quantified simple actives (which are included as fillers, mean = 1.27), suggesting that inverse scope reading is more available under the environment of adverbial clauses. Putting aside the factors we controlled in the stimuli and just focusing on the target sentences and contexts which favor inverse scope (see the numbers that are shaded with gray in Tables 2, 3), we can see that the mean ratings of the sentence-context matching pairs would be higher—close to or over 3, very similar to the mean rating of doubly-quantified PP datives, which is just 3.33. Additionally, about one-fifth of the participants (18 out of 98) consistently gave a rating higher than 2 on sentence-context matching pairs for all target sentences they were asked; it means that the inverse scope reading is fully available for these participants regardless of adverbial clause type, presence of aspect marker or embedded verb type. Therefore, this average range of 2.5 to 3.0 acceptability ratings in this study, we claim, characterizes that inverse scope reading is available for the doubly-quantified simple transitives under adverbial clauses.

The mean ratings on context-sentence pairs for doubly-quantified transivites under adverbial clauses are much higher than on context-sentence pairs for matrix simple transivites. The results support Scontras et al. (2016) and Grano (2017)’s conjecture that conditional clause environment contributes to scope ambiguity, and provide evidence against Aoun and Li (1989)’s claim that (4) is purely scope-rigid, just as the matrix simple transitives.

The fact that simple actives could have inverse scope readings under an adverbial clause environment but not under a matrix clause environment, then suggests that clause environment matters here. The results regarding quantifier scope in adverbial clauses provide an empirical challenge to the long-standing scope-rigidity view of Mandarin, which argues for a one-to-one mapping between the linear order of quantificational phrases and scope interpretation. Previous approaches which inherit the idea of isomorphism, such as the Isomorphism Principle (10) by Huang (1982),^12^ and the Linearity Principle (11) by Lee (1986, 1991), cannot account for the availability of inverse scope in Mandarin adverbial clauses.

(10)  General condition on scope interpretation (Huang, 1982:220, example 70)
     Suppose A and B are both QPs (quantifier phrases) or both Q-NPs or Q-expressions; then if A c-commands B at S-Structure (SS), A also c-commands B at the Logical Form (LF).

(11)  General Condition on Scope Interpretation (Lee, 1986, p.142)
     Suppose A and B are both QPs or both Q-NPs or Q-expressions, then.

(i) if A asymmetrically commands B at SS, A has scope over B at LF;
(ii) if A and B command each other and A precedes B at SS, A has scope over B at LF.

(A commands B iff neither dominates the other and the first minimal clause dominating A also dominates B.)

Since the existential quantificational phrase inside the adverbial clauses does not have a mutual c-commanding relation with the universal quantificational phrase inside the adverbial clause, these approaches would all wrongly predict that the target sentences are purely scope-rigid with surface scope only.

Similarly, the doubly-quantified transitives under adverbial clauses are wrongly predicted to show surface scope only under the Scope Principle (12) and Minimal Binding Requirement (13) proposed by Aoun and Li (1989, 1993).

(12)  Scope Principle.
     A quantifier A has scope over a quantifier B in case A c-commands a member of the chain containing B. (Aoun and Li, 1989, example 20).

(13)  Minimal Binding Requirement.
     Variables must be bound by the most local potential antecedent ( $\bar{A}$ -binder). (Aoun and Li, 1989, example 12).

The scopal contrast between unambiguous case (14a) and unambiguous case (14b) can be represented as in the following example wherein x1 and x2 are the variables of quantificational phrase 1 (QP1) and quantificational phrase 2 (QP2) after quantifier raising. According to Aoun and Li (1989), the availability of inverse scope is related to the existence of a trace, more precisely, whether there is a trace of the higher QP over which the lower QP can have a wide scope.

(14)  a. QP_2_ *x*_2_ QP_1_ *x*_1_ *t_2_* (ambiguous)
b. QP_1_ *x*_1_ QP_2_ *x*_2_ (unambiguous) (Aoun and Li, 1989, example 23).

There is no overt movement involved in the simple actives under doubly-quantified adverbial clauses unless we assume that the doubly-quantified simple actives under adverbial clauses have subject raising process like English matrix actives do (15) to give universal quantificational phrase the pathway to scope over the trace *t*_i_ after quantifier raising to the edge of VP. However, the assumption would require us to come up with some magical *ad hoc* techniques to assure that the subject-raising process is only applicable for simple transitives under an adverbial clause in Mandarin, but not applicable for matrix simple transitives in Mandarin.

(15)  a. *[_I″_ two men_i_ [_I′_ I [_VP_ *t*_i_ found every clue]]] (the doubly-quantified simple actives under adverbial clauses)
     b. [_I″_ someone_i_ [_I′_ I [_VP_ *t*_i_ loves everyone]]] (English; Aoun and Li, 1989, example 36).

The results also propose challenges to previous approaches on quantifier scope that consider thematic hierarchy as a factor in the scope interpretation. Lee (1991: 204) proposes to have (16) in addition to the Linearity Principle (11). He attributes the scope ambiguity to “joint effects of the linearity principle and a thematic hierarchy” following the proposal made by Xu and Lee (1989).

(16)  Thematic Hierarchy (Xu and Lee, 1989; Lee, 1991)
     (Group A): Agent, Location, Source, Goal.
     (Group B): Theme, Patient, Factitive (Narrow Scope Thematic Roles).

According to Xu and Lee (1989), a quantificational phrase carrying the thematic role in Group A is more likely to take wide scope over a quantificational phrase carrying the thematic role in Group B. When the scope predicted by the thematic hierarchy in (16) conflicts with the scope predicted by the linear order, scope ambiguity arises.

For example, in a PP dative construction: verb – direct object- *gei*- indirect object, thematic hierarchy in (16) predicts that the indirect object (the goal) to take wide scope over the direct object (theme), but the linear order predicts direct object scoping over the indirect object. Therefore, the quantifier scope ambiguity of doubly quantified PP dative constructions (9) is expected under this account.

However, Xu and Lee (1989) and Lee (1991)‘s account fails to take the argument structures into consideration. The thematic hierarchy assumed in (1) is dubious under the widely held hierarchy of thematic roles in the literature: Agent > Theme > Goal > Location > Source (see discussions about thematic structures at Carrier-Duncan, 1985; Baker, 1988; Belletti and Rizzi, 1988; Larson, 1988, 1990, 2014; Jackendoff, 1990). This account also assumes scope ambiguity comes from the joint influence of word order and thematic hierarchy assumed in (16), but not from quantifier raising. It conflicts with the argument that quantifier raising exists in Mandarin and contributes to scope interactions in Mandarin, just as what quantifier raising would do in other languages (Lin, 2003b, 2013, 2015; Grano, 2017 among others). For example, in (17) where the universal quantifier embedded in the PP scopes over the existential quantifier, similar to the inverse linking cases studied in the literature (see more discussion on inverse linking and quantifier raising in Bobaljik and Wurmbrand, 2012; Antonyuk, 2019).

(17)  *Zhìshǎo wǔ-wèi měi-yī-zhōu yìhuì de yìyuán huì zhīchí zhè-gè-tí’àn*.
     at.least five-_CL_ every-one-state congress link congressman will support this-_CL_-proposal.
     a. at least 5 > ∀: ‘There are at least five of the congressmen of every state congress supporting this proposal.’
     b. ∀ > at least 5: ‘For each state congress, at least five of the congressmen will support this proposal.’ (Lin, 2013: ex.7)

Moreover, the joint account of the thematic hierarchy in (16) and the linear word order for scope interpretations, would wrongly predict (18) being scope frozen, as both the thematic hierarchy in (16) and the linear word order predict *two students* (agent) scope over *every problem set* (theme). But, our results suggest that the inverse scope reading of sentences like (18) is available to a large portion of participants. 71% of participants (70 out of 98 participants) gave a rating higher than 3 at least once for such sentences.”

(18)  *Suīrán*     *liǎng-gè-xuésheng*     *dádu-le*
     although     two-clf-student answer-correct(-asp)
     *měi-dào-tí*     *Hǎidiànduì*     *háishi*     *méiyǒu*
     every-clf-problem.set     Haidian.team     still     not
     *rùxuǎn*     *àoshù*     *jíxùnduì*
     get.selected     Math.Olympiad     training.program

‘Although two students answer(ed) every problem set correctly, Haidian team still did not get selected to join the Math Olympiad training program.’

(*Context given along with this sentence*: The rules of the qualification exam for the Math Olympiad training team are: each team consists of three members and there are three problem sets in total; for a team, each of the three problem set needs to be answered by two members of that team and a team is possible to join the Math Olympic training program when that team gives correct answers for all three problem sets. Three members of the Haidian team are Xiao Wang, Xiao Jiang and Xiao Zheng. Xiao Wang and Xiao Jiang answered the first two problem sets correctly. Xiao Jiang and Xiao Zheng answered the last problem set correctly. The Haidian team met the selection criteria, but they were not selected to join the training program. Xiao Wang, Xiao Jiang, and Xiao Zheng were very sad.)

As shown above, the results suggest that we need more refined accounts beyond the existing syntactic accounts (Huang, 1982; Aoun and Li, 1989) or semantic accounts (Xu and Lee, 1989 and Lee, 1991). The results call for rethinking the long-standing dichotomy view of quantifier scope in Mandarin Chinese and call for rethinking the existing approaches to scope interpretations. Our results demonstrate that inverse scope of simple transitives under adverbial clauses is available in Mandarin Chinese, although Mandarin simple transitives are claimed to be scope rigid. Our conjecture for the different scope behaviors between simple matrix transitives and simple transitve under adverbial clauses is that clause size matters for scope permutation, an idea that has been noted in Lin (2013), Grano (2017), and Wu et al. (2018) and is worthy of further study.

The availability of the inverse scope reading of doubly-quantified simple transitives under adverbial clauses calls for a modification of the theoretical claim that scope rigidity is a property of Mandarin Chinese. Figures 5, 6 show that a large number of participants gave very high ratings for the context-sentence matching pairs in the stimuli, which would be unexpected if scope-rigidity/fluidity were a language property and Mandarin was a scope-rigid language. Furthermore, the results presented in Figures 5, 6 also provide evidence against the dichotomy of scope interpretations at a specific syntactic construction level. If scope-rigidity were the property of a specific syntactic construction and adverbial clause environment were considered as a pure scope-rigid or scope-fluid environment, we would not expect a bimodal distribution of the data at all. In fact, we found that about half of the 98 participants think that the target adverbial clauses with the linear order of ∃ over ∀ match an ∀ over ∃ scenario, while the other half of the participants disagree with it. The bi-modal distribution of the data suggests that it is more than the dichotomy of scope interpretations that affects the scope interpretations.

Bimodal distribution of scope interpretations has been observed in previous experimental scope studies. Scontras et al. (2016) report that half of the 30 surveyed English speakers find sentences like (19) ambiguous while the other half do not.

(19)  There is a shark that attacked every pirate. (Scontras et al., 2016, ex. 13)

According to Scontras et al. (2016), two different grammars of relativization in English is a potential explanation for the observed bi-modal distribution of scope interpretations. Scontras et al. (2016) argue that, if English restrictive relative clauses, like what Hulsey and Sauerland (2006) suggested, are structurally ambiguous between the head-internal, raising structure and the matching structure then it is likely that some speakers apply one structure whereas other speakers apply another.^13^

Experimental studies on scope interpretations of other languages also observed bimodal distribution on acceptance of inverse scope interpretations. Han et al. (2007) found that only about half of the 160 surveyed Korean speakers allow negation to take scope over a quantificational phrase in the object position for Korean sentences like (20). Based on the finding, Han et al. (2007) argued that there might be two populations of native speakers that have two different grammars: one with a verb-raising mechanism and one without.

(20)  *Khwukhi*     *Monste-ka*     *motun*     *khwukhi-lul*     *an mek-ess-ta.*
       Cookie     Monster-nom     every     cookie-acc neg eat-pst-decl.
       ‘Cookie Monster did not eat every cookie.’ (Han et al., 2007, ex. 54b).

Both Han et al. (2007) and Scontras et al. (2016) suggest the bimodal distribution of the acceptance of inverse scope readings may come from structural ambiguity in speakers’ grammar. Following this suggestion, the bimodal distribution we observed in the present study could potentially come from the structural ambiguity of adverbial clauses. Adverbial clauses have been argued to have dual status: central adverbial clauses vs. peripheral adverbial clauses, where the former has less root functional projections available, but the latter has more root functional projections available (See Haegeman, 2002 and subsequent works for the dual status discussions on English and German adverbial clauses, and Lu 2003, 2008; Pan and Paul, 2018, Wei, 2018, Wei and Li, 2018 for the related discussions on Mandarin). It is then possible that some speakers apply the peripherical structure while others apply the central structure of an adverbial clause and correspondingly give divergent ratings on the availability of inverse scope reading. If it were the case, we would predict that doubly-quantified transitives under the peripheral adverbial clause status have more comparable to the matrix doubly-quantified transitives with respect to scope interpretations. But to testify to such prediction, we need further study to give clear and unified criteria on the categorization of peripheral versus central adverbial clauses, which we do not have at this moment due to conflicting claims in the literature.

Additionally, the results echo Zhou and Gao (2009)’s generalization that the lexical information of verbs may have effects on scope judgments. Zhou and Gao (2009) found that, in an offline judgment task, surface scope reading is more readily accessible than the inverse scope reading for the action verbs, and the locative verbs, while for the psych verbs, the surface scope reading and the inverse scope reading are equally accessible. With respect to thematic relations between subjects and objects, the durative verbs and resultative verbs used in the present study are comparable to the locative verbs and action verbs used by Zhou and Gao (2009) respectively. In the present study, when the verb is a durative verb, the quantified subject expresses a theme and the quantified object expresses a location; when the verb is a resultative verb, the quantified subject expresses an agent and the quantified object expresses a theme. Although the present study does not directly compare the influence of verb types on which scope reading is more accessible, we observed a similar pattern as Zhou and Gao (2009) mentioned in their paper: when the verbs are locative verbs, the inverse scope reading was judged with a bit higher average rating for locative verbs than for action verbs. Participants in the present study gave an average rating of 2.82 on the context-sentence pair for the target sentences with durative verbs (*xiézhù* ‘assist’, *bùzhì* ‘decorate (a room)’, *kānshǒu* ‘guard’) and an average rating of 2.43 on the context-sentence pair for the target sentences with resultative verbs (*chīdiào* ‘eat up’, *dáduì* ‘answer (questions) correctly’, *dāchū* ‘build up’). It suggests that participants in the present study judged inverse scope readings are more accessible for the target sentences with durative verbs than for the target sentences with resultative verbs. In other words, the thematic information of the verbs does affect the scope interpretations.

We also observed that the combination of verbs and aspect marker *le* could have a significant effect on the context-sentence ratings. Our results show that when the verbs are resultative verbs, higher average ratings on context-sentence compatibility were found for the sentences with aspect marker *le* than without *le*. The pattern is reversed when the verbs are durative verbs. At this moment, the inter-dependence between verb type and the presence of *le* remains to be accounted for, although we may attribute the observed inter-dependence pattern to the sentence naturalness and frequency of the combination of verbs and aspect markers. As mentioned in the experiment design, when the embedded verbs are resultative verbs (*chīdiào* ‘eat up’, *dáduì* ‘answer (questions) correctly’, *dāchū* ‘build up’), they are more naturally compatible and more frequently used with the aspect marker *le*, and no other temporal adverb or temporal phrase is necessarily required. Durative verbs, like *xiézhù* ‘assist’ or *kānshǒu* ‘guard’, tend to take temporal phrases/ adverbs when combined with *le*. For instance, *ta yijing kānshǒu-le san-tian de damen* ‘he has already guarded the front door for 3 days’, in which *yijing* ‘already’ and *san-tian* ‘3 days’ indicate how long the state of guarding door has been.

The effect of the internal structure of quantificational phrases is also shown in the results. When the existential quantificational phrase is in the form of *yi* ‘a/one’ + classifier + noun, the context-sentence rating is generally lower, suggesting that *yi* ‘a/one’ is more unfavorable for existential quantificational phrases taking narrow scope. This finding echoes the observations in the literature that existential quantifier *yi* tends to be interpreted as referential/ specific by both children and adults and then be assigned with a wide scope reading (Lee, 1986; Chien, 1994; Liu, 1997; Scontras et al., 2017; Yang and Wu, 2020). On the other hand, it is noticeable that *yi* ‘a/one’ does not correspond to a lower context-sentence rating in the *yàoshì* conditional clause. It could be the case that the hypothetical environment of conditional clauses overriding the preferred referential interpretation of *yi*; as a result, the quantified subject in the form of *yi* + classifier+noun gets more availability to have scope permutation with the universal quantificational phrase in the embedded object position. This interaction between the internal structure of quantificational phrases and adverbial clause type again suggests that scope interpretations may be constrained by factors including semantics, pragmatics, and processing strategy.

In this section, we discussed the implications of the results. We showed that the previous proposals to Mandarin quantifier scope interpretation have difficulty in accounting for the availability of inverse scope readings in doubly-quantified simple transitives under Mandarin adverbial clauses. The results call for a refined scope account that dispenses with the isomorphic view of Mandarin quantifier. Bimodal distribution on the on the acceptance of inverse scope readings draw our attention to the intra-participant variance, which again calls for rethinking the dichotomy view of quantifier scope in languages. In the end, the observed other factors that may affect scope behaviors, including verb type, and numbers, draw our attention on the influence of lexical information on quantifier scope interpretations.

## 5. Conclusion and future work

In this paper, we have examined the scope behaviors in Mandarin doubly-quantified transitives under adverbial clauses (*suīrán* ‘although’*, yàoshì* ‘if’ *and yīnwèi* ‘because’) through an untimed, offline experiment using Truth-Value Judgment Task. The results of this experiment demonstrate that i) inverse scope readings are judged to be available in Mandarin doubly-quantified transitives under adverbial clauses but a bimodal data distribution is observed; ii) the ratings on the compatibility of inverse-scope context and target sentences do not vary significantly based on the type of adverbial clauses; iii) verb types and the presence of aspect maker *le* are inter-dependent and together have a significant effect on scope interpretations; (iv) participants tend to give a lower rating on the context-sentence compatibility when the existential quantificational phrases are *yi* (‘a’/‘one’) + classifier + noun compared to *liǎng* (‘two’)*/sān* (‘three’) + classifier + noun.

These experimental results suggest the following theoretical implications. First, scope rigidity as a language parameter of Mandarin is not a sufficient account for the results observed in the present study. Second, the dichotomy of scope interpretations at a syntactic construction level is not adequate to explain scope behaviors either, as bimodal data distribution was found within the same syntactic environment, which is little surveyed in the literature. Third, the speaker variations, especially the bimodal distribution of scope interpretations, may come from the complexity of the examined syntactic structures (e.g., central versus the peripherical status of adverbial clauses), which calls for more research on how structural ambiguity affects scope interpretations. Fourth, lexical information of verbs and numbers should also be taken into consideration in the future for discussions on scope interpretations.

Given that the reported findings are collected from 98 native speakers, a decent number of samples, and participants were given different target sentences randomly, we can reasonably assume that the differences in ratings on the context-sentence pairs result from the different factors that were manipulated in the study. Meanwhile, we admit there are some limitations of the present study. One limitation is the present design did not include a controlled group, which may degrade the validity of the findings. Another limitation is that the present study used written narratives to present an inverse-scope reading scenario and asked participants to judge if the written target sentences match the scenario. The findings of the present study are then limited to the scope interpretations of written Mandarin, with factors like prosody uninvestigated. We also admit that the written narratives used in the present study to represent the contexts were comparably lengthier than the ones used by Zhou and Gao (2009), which may cause extra reading time and processing costs for participants. It is possible that participants in this study found the narrative interesting to read but got distracted by trivial information and then missed the most significant information to make the most faithful scope judgments. At this moment, we have no direct comparisons among different ways of presenting contexts: detailed written narrative, condensed written narratives, oral or audio narratives, act-out storytelling, or pictures, and yet no clear answers about which way is the best way to present a context for a Truth-Value Judgment Task. We will leave the evaluation of methodologies to future work.

With these limitations noted, a number of directions for future study can be pursued. One direction is to add a controlled group to the experiment design, take further measures to exclude the potential confounding factors in the acceptability experiment, and then experimentally test the scope interpretations using oral materials (Ionin and Luchkina, 2018; Kırcalı et al., 2021) or pictures (Scontras et al., 2017) or condensed written narratives (Zhou and Gao, 2009) as materials. Another direction is to change the offline judgment task into an online judgment task (Anderson, 2004) to investigate the online processing of scope interpretations by setting a limited time duration for the experiment and recording participants’ response times across conditions. The present study investigates the scope of interactions between bare-numeral quantificational phrases and strong distributive-universal quantificational phrases, but not all quantifiers behave with the same properties (Beghelli and Stowell, 1997). Therefore, another direction to pursue is to manipulate the types of quantifiers and experimentally investigate if scope interactions are different from the results we observed in the present study.

As the present study argues against the scope rigidity tag on Mandarin, it would be also interesting to extend the present study to other non-matrix syntactic environments in Mandarin, for example, testing the scope interpretations in Mandarin embedded nonfinite clauses, where, the inverse scope reading is available according to Lin (2013), but Gan (2021) disagrees. From a crosslinguistic perspective, it would be fruitful to extend the study to experimentally test the doubly quantified transitives under adverbial clauses in some other claimed-to-be “scope-rigid” languages.

## Data availability statement

The original contributions presented in the study are included in the article/Supplementary material, further inquiries can be directed to the corresponding author.

## Ethics statement

The studies involving human participants were reviewed and approved by Stony Brook University, the Office of Research Compliance, IRB2019-00115. Written informed consent for participation was not required for this study in accordance with the national legislation and the institutional requirements.

## Author contributions

HW contributed to the conception and design of the study, organized the database, performed the statistical analysis, wrote the first draft of the manuscript, and worked on the manuscript revision, read, and approved the submitted version.

## Funding

This study is supported by the Faculty Development Fund awarded to the author by the School of Modern Languages at Georgia Institute of Technology.

## Conflict of interest

The author declares that the research was conducted in the absence of any commercial or financial relationships that could be construed as a potential conflict of interest.

## Publisher’s note

All claims expressed in this article are solely those of the authors and do not necessarily represent those of their affiliated organizations, or those of the publisher, the editors and the reviewers. Any product that may be evaluated in this article, or claim that may be made by its manufacturer, is not guaranteed or endorsed by the publisher.

## Footnotes

^1^It has been claimed that Mandarin sentences with indefinite (i.e., ‘number + classifier + noun’) subjects tend to have *you* ‘have, exist’ at the beginning of sentences otherwise it is not natural (Chao, 1968; Li and Thompson, 1981; among others). However, *you*-less sentences with indefinite subjects are considered natural and used in experimental studies as well (Fan, 1985; Su, 2001; Chen, 2004; Jiang, 2012; section 3.2.3, among others).

^2^Thanks to a reviewer for pointing out a potential bias of lexical items used in the stimuli. When it comes to a policeman guarding every exit, the inverse scope reading would be for every exit there is one different policeman to guard it. To clarify, the inverse scope reading does not refer to the logically impossible reading where a single police guard is at each exit. See more insightful discussions on the contrast between *each* and *every* at Reinhart (1997: 369) and Beghelli and Stowell (1997). In fact, the inverse scope reading of a policeman guarding every exit is pragmatically preferred, as we would prefer to have different policeman guards at different exits for better security reasons. To test if inverse scope reading is available for other contexts (i.e., contexts that do not have the pragmatic preferences for inverse scope reading), we varied the lexical items used in the stimuli and considered different sets of stimuli as random effects when computing statistical analysis, to minimize the possible lexical bias towards the overall results. In the stimuli, we used six different sets of lexical items, such as, students answering problem sets, dogs eating cakes.

^3^Thanks to a reviewer for pointing out the debates on attachment positions of adverbial clauses. It is debated in the literature whether an adverbial clause is positioned in a coordination position of a main clause or positioned in a Specifier position of the DiscuourseP in the main clause (see Wei, 2018 for insightful discussions on the attachment position of Mandarin adverbial clauses; see Lund and Charnavel, 2020 on English adverbial clauses). Here we did not specify the syntactic attachment positions of these adverbial clauses, but rather just assume a linear order between the adverbial clause and main clauses, which is all adverbial clauses in the stimuli are positioned sentence-initially with the order of adverbial clause - main clause.

^4^One may refer to such verbs as a phrase with the form of V+ complement, but since these compounds behave no differently from a disyllabic verb, here we consider the V-V compounds as verbs, like what Zhu (1982:126-127) has suggested.

^5^In the stimuli, all doubly-quantified simple transitives under adverbial clauses are strictly lexically matched in the same set. The matrix clauses following the adverbial clauses have been varied a bit to make the target sentence more natural.

^6^A reviewer raised concerns about whether the judgments of the participants were based on their correct understanding of the instructions as well as the context, and suggested identifying and removing the problematic scores. We appreciate the reviewer’s questions and admit that there might be some participants who submitted the ratings randomly. However, it is not clear how to judge an acceptability rating being problematic. A participant might read all the instructions and target sentences, and choose a rating of 2 for all conditions, while another participant just rated the sentences blindly, and also chose a rating of 2 for all conditions. Some measures were taken to ensure participants rate sentences based on their language intuition and their perception of the instructions: (i) Participants were given a practice session to get familiar with the format of the judgment task and the task only included 24 questions with no time pressure to complete; (ii) different colors and formats were used to represent target sentences and instructions to raise participants’ attention (Figure 1). Moreover, a large number of participants were recruited to help balance out the potential confounding issue of some participants not paying attention, as we generally assume that the majority of 98 participants would do this task carefully and faithfully. When performing statistical analysis, by-participant and by-set variations were taken into consideration to minimize the potential effects of some participants giving ratings randomly and blindly. In follow-up experiments, we will take further steps to check if participants pay close attention to the instruction. For example, include some reading comprehension questions as fillers in the experiment where a given context is about how a group of students split the cost of an event, and the target sentence gives a false statement about the situation. If participants give a high rating on this kind of question, then we would exclude his/her/their data.

^7^We thank an anonymous reviewer for introducing relevant references to us and suggesting we further evaluate and justify the choice of random effect structure used in the study. We also checked the singularity and PCA results of three random slope models, and both models were detected with singularity and zero variance components.

- fm.full.1.slope < − *lmer(response ~ adverbial_type + presence_le + (1 + adverbial_type|participant) + (1 + adverbial_type|set),data = data ,REML = FALSE)*
- fm.full.2.slope < − *lmer(response ~ adverbial_type + presence_le + (1 + adverbial_type|participant) + (1 + adverbial_type|set), data = data,REML = FALSE)*
-    fm.full.3.slope < − lmer(response ~ adverbial_type + presence_le + (1 + adverbial_type + presence_le|participant) + (1 + adverbial_type + presence_le|set),data = data,REML = FALSE).

^8^For example (7) and the following examples of stimuli and fillers, we included pinyin with tones, English gloss and English translations for sentences, and English translations for contexts, to save some space. A full list of stimuli with Chinese characters and a detailed gloss for contexts can be found in the Supplementary Materials.

^9^Some speakers may find sentences like (9) unnatural because they prefer to have a double object construction like [Subject Verb-*gei* + indirect object + direct object structure], as noted in Gan (2021: 71), although similar sentences like (9) have been used as examples in literature (Lin, 2013: 278). It is possible that these speakers may assign a low score due to its ungrammaticality but not because of its (inverse) scope. We do not know how many participants in the present study share this kind of sentence judgment, but we do take by-participant as a random intercept when computing statistical analysis.

^10^The three models are:

- *fm.full1 < − lmer(response ~ adverbial_type + (1|participant) + (1|set), data = data, REML = FALSE)*;
- *fm.full2 < − lmer(response ~ presence_le + (1|participant) + (1|set), data = data, REML = FALSE)*;
- *fm.reduced < − lmer(response ~ (1|participant) + (1|set), data = data, REML = FALSE).*

^11^Z-score normalization of ratings are performed by participant. For a participant, a z-normalized rating is calculated through the formula in (i). (i) z-normalized rating = (raw rating – mean of all ratings that participant gave) /standard deviation of all ratings that participant gave.

^12^The general scope condition proposed in Huang (1982) was termed as "Isomorphism Principle" in Aoun and Li (1989).

^13^Note that some studies proposed a pure raising analysis of English relative clauses (Bianchi, 2002a,b). Thanks to a reviewer for suggesting the references.

## References

[ref1] Anderson C. (2004). The structure and real-time comprehension of quantifier scope ambiguity. Dissertation. Chicago (IL): Northwestern University.

[ref2] Antonyuk S. (2019). Quantifier scope in Russian. Glossa 4, 1–27. doi: 10.5334/gjgl.562

[ref3] Aoun J. Li Y.-H. A. (1989). Scope and constituency. Linguist. Inq. 20, 141–172.

[ref4] Aoun J. Li Y.-H. A. (1993). Syntax of scope. Cambridge, MA: MIT Press.

[ref1011] Baker M. C. (1988). Incorporation: A theory of grammatical function changing. Chicago, IL: University of Chicago Press.

[ref5] Bates D. Mächler M. Bolker B. Walker S. (2015a). Fitting linear mixed-effects models using lme4. J. Stat. Softw. 67, 1–48. doi: 10.18637/jss.v067.i01

[ref6] Bates D. Kliegl R. Vasishth S. Baayen H. (2015b). Parsimonious mixed models. ArXiv [Preprint].

[ref7] Beghelli F. Stowell T. (1997). “Distributivity and negation: the syntax of each and every” in Ways of scope taking. ed. Szabolcsi A., vol. 65 (Netherlands: Springer), 71–107.

[ref8] Belletti A. Rizzi L. (1988). Psych-verbs and θ-theory. Nat. Lang. Linguist. Theory 6, 291–352. doi: 10.1007/BF00133902

[ref9] Bianchi V. (2002a). Headed relative clauses in generative syntax. Part I. Glot Int. 6, 197–204.

[ref10] Bianchi V. (2002b). Headed relative clauses in generative syntax. Part II. Glot Int. 6, 1–13.

[ref11] Bobaljik J. D. Wurmbrand S. (2012). Word order and scope: transparent interfaces and the 3/4 signature. Linguist. Inq. 43, 371–421. doi: 10.1162/LING_a_00094

[ref12] Brendel C. (2019). An investigation of numeral quantifiers in English. Glossa 4:104. doi: 10.5334/gjgl.391

[ref13] Bross F. (2019). Using mixed effect models to analyze acceptability rating Data.Version 1.0. Mimeo. Available at: www.fabianbross.de/mixedmodels.pdf (Accessed April 10, 2023).

[ref1010] Carrier-Duncan J. (1985). Linking of thematic roles in derivational word formation. Linguistic Inquiry 16, 1–34.

[ref1001] Chao Y. (1968). A grammar of spoken Chinese. Berkeley, CA: University of California Press.

[ref1003] Chen P. (2004). Identifiability and definiteness in Chinese. Linguistics 42, 1129–1184.

[ref14] Chen Y. (2020). “An experimental investigation of reconstruction effects of the head quantifier phrase in Chinese relative clauses.” in Paper presented at the 32nd North American Conference on Chinese Linguistics, a conference held at the University of Connecticut, Storrs (online), September 18–20, 2020.

[ref15] Chien Y. C. (1994). “Structural determinants of quantifier scope: an experimental study of Chinese first language acquisition” in Syntactic theory and first language acquisition: Cross-linguistic perspective Vol. 2. eds. Lust B. Hermon G. (Hillsdale, NJ: Lawrence Erlbaum), 391–415.

[ref16] Chien Y. C. Wexler K. (1989). “Children’s knowledge of relative scope in Chinese” in Papers and report in child language development number 28. eds. Clark E. V., E. O. Bratt, S. L. Cheung, and B. McElhinny (Stanford, CA: Stanford University), 72–80.

[ref17] Crain S. McKee C. (1985). “The acquisition of structural restrictions on anaphora” in Proceedings of NELS 15. eds. Berman S. Choe J.-W. McDonough J. (Amherst: University of Massachusetts, GLSA), 94–110.

[ref18] Crain S. Thornton R. (1998). Investigations in universal grammar: A guide to experiments on the acquisition of syntax and semantics. Cambridge, MA: MIT Press.

[ref1013] Fan J. (1985). Wuding NP zhuyu ju [‘Sentences with indefinite NP subject’]. Zhongguo Yuwen [‘Chinese Language and Writing’] 5, 321–328.

[ref19] Gan T. (2021). Quantifiers and scope ambiguity in mandarin: theoretical and experimental perspectives. Dissertation. [Hongkong (China)]: City University of Hong Kong.

[ref20] Gibson E. Thomas J. (1999). Memory limitations and structural forgetting: the perception of complex ungrammatical sentences as grammatical. Lang. Cogn. Process. 14, 225–248. doi: 10.1080/016909699386293

[ref21] Grano T. (2017). Finiteness contrasts without tense? A view from mandarin Chinese. J. East Asian Linguis. 26, 259–299. doi: 10.1007/s10831-017-9159-8

[ref22] Haegeman L. (2002). Anchoring to speaker, adverbial clauses and the structure of CP. Georgetown University working papers in theoretical linguistics 2: 117–180.

[ref1005] Haegeman L. (2006). Clitic climbing and the dual status of sembrare. Linguistic Inquiry 37, 484–501.

[ref1006] Haegeman L. (2012). Adverbial clauses, main clause phenomena, and composition of the left periphery: The cartography of syntactic structures (Vol. 8). Oxford, UK: Oxford University Press.

[ref23] Han C. H. Lidz J. Musolino J. (2007). Verb-raising and grammar competition in Korean: evidence from negation and quantifier scope. Linguist. Inq. 38, 1–47. doi: 10.1162/ling.2007.38.1.1

[ref25] Harris J. A. Clifton C. Jr. Frazier L. (2013). Processing and domain selection: quantificational variability effects. Lang. Cogn. Process. 28, 1519–1544. doi: 10.1080/01690965.2012.679663 25328262 PMC4200393

[ref1008] Heizmann T. (2007). (Un)Frozen Scope in English and German Double Object Constructions, University of Massachusetts Occasional Papers in Linguistics. Vol. 33. Available at: https://scholarworks.umass.edu/umop/vol33/iss1/6 (Accessed January 20, 2023).

[ref26] Hoji H. (1985). Logical form constraints and configurational structures in Japanese. Doctoral dissertation. [Seattle (WA)]: University of Washington.

[ref27] Huang S. F. (1981). On the scope phenomena of Chinese quantifiers. J. Chin. Linguist. 9, 226–243.

[ref28] Huang C.T. J. (1982). Logical relations in Chinese and the theory of grammar. Ph.D. thesis. MIT.

[ref1012] Hulsey S. Sauerland U. (2006). Sorting out Relative Clauses. Nat. Lang. Semantics 14, 111–137. doi: 10.1007/s11050-005-3799-3

[ref29] Ionin T. Luchkina T. (2018). Focus on Russian scope: an experimental investigation of the relationship between quantifier scope, prosody, and information structure. Linguist. Inq. 49, 741–779. doi: 10.1162/ling_a_00288

[ref30] Jackendoff R. (1990). On Larson’s treatment of the double object construction. Linguist. Inq. 21, 427–456.

[ref1004] Jiang L. (2012). “Nominal structure and language variation,” in Doctoral Dissertation. (Cambridge MA: Harvard University).

[ref31] Kırcalı C.E. Uzun İ.P. Aydın Ö. (2021). “Syntactic and prosodic processing of quantifier ambiguity in Turkish.” in Proceedings of the Workshop on Turkic and Languages in Contact with Turkic, vol. 6.

[ref32] Larson R. K. (1988). On the double object construction. Linguist. Inq. 19, 335–391.

[ref33] Larson R. K. (1990). Double objects revisited: reply to Jackendoff. Linguist. Inq. 21, 589–632. doi: 10.2307/4178697

[ref34] Larson R. K. (2014). On shell structure. London, UK: Routledge.

[ref35] Lee T. H.K. (1986). Studies on quantification in Chinese. Dissertation. [Los Angeles (CA)]: University of California, Los Angeles.

[ref36] Lee T. H. K. (1989). The role of linear order in the acquisition of quantifier scope in Chinese. CUHK Papers Linguist. 1, 18–45.

[ref37] Lee T. H. K. (1991). “Linearity as a scope principle for Chinese: the evidence from first language acquisition” in Bridges between psychology and linguistics. eds. Napoli D. Kelg J. (Hillsdale, NJ: Erlbaum), 183–204.

[ref38] Lee T. H. K. (2002). Two types of logical structure in child language. J. Cogn. Sci. 3, 155–182.

[ref39] Lee T. H. K. Yip V. Wang C. (1999). Inverse scope in Chinese-English interlanguage. Lingua Posnaniensis 41, 49–66.

[ref1002] Li C. Thompson S. (1981). Mandarin Chinese: a functional reference grammar. Berkeley, CA: University of California Press.

[ref41] Lin J. W. (2003a). Aspectual selection and negation in mandarin Chinese. Linguistics 41, 425–459. doi: 10.1515/ling.2003.015

[ref42] Lin J. W. (2003b). Temporal reference in mandarin Chinese. J. East Asian Linguis. 12, 259–311. doi: 10.1023/A:1023665301095

[ref43] Lin T. H. J. (2013). “QR and finiteness” in Deep insights, broad perspectives: Essays in honor of Mamoru Saito. eds. Miyamoto Y. D., D. Takahashi, H. Maki, M. Ochi, K. Sugisaki, and A. Uchibori (Tokyo: Kaitakusha), 275–291.

[ref44] Lin T. H. J. (2015). Tense in mandarin Chinese sentences. Syntax 18, 320–342. doi: 10.1111/synt.12032

[ref45] Liu F. H. (1997). Scope and specificity. Amsterdam, Netherlands: John Benjamins.

[ref46] Liu Y. Wu H. (2016). Quantifier scope in mandarin Ditransitives. Presented at NACCL 28 (28th North American Conference on Chinese Linguistics), Utah, USA.

[ref47] Lu P. (2003). La subordination adverbiale en chinois contemporain. Dissertation. [Paris (France)]: University Paris 7.

[ref48] Lu P. (2008). Les phrases complexes en chinois. Beijing: Foreign Language Press.

[ref49] Lund G. Charnavel I. (2020). The syntax of concessive clauses: evidence from exempt anaphora. Univ. Pennsylvania Work. Papers Linguist. 26, 159–168.

[ref50] Pan V. J. Paul W. (2018). The syntax of complex sentences in mandarin Chinese: a comprehensive overview and analyses. The syntax of complex sentences in Chinese. Linguist. Anal. 42, 63–161.

[ref1007] R Development Core Team (2019). R: A Language and Environment for Statistical Computing. Vienna, Austria: R Foundation for Statistical Computing. Available at: https://www.R-project.org/ (Accessed December 20, 2019).

[ref51] Reinhart T. (1997). Quantifier scope: how labor is divided between QR and choice functions. Linguist. Philos. 20, 335–397. doi: 10.1023/A:1005349801431

[ref52] Scontras G. Polinsky M. Tsai C. Y. E. Mai K. (2017). Cross-linguistic scope ambiguity: when two systems meet. Glossa 2:36. doi: 10.5334/gjgl.198

[ref53] Scontras G. Tsai C. Y. E. Mai K. Polinsky M. (2016). “Revisiting inverse scope: an experimental study of Chinese and English” in Proceedings of Sinn un Bedeutung 18th ed. eds. Etxeberria U. Fălăuș A. Irurtzun A. Leferman B. (Vitoria-Gasteiz, Spain: University of the Basque Country (UPV/EHU)), 396–414.

[ref54] Su Y.C. (2001). Scope and specificity in child language: A cross-linguistic study on English and Chinese. Dissertation. [College Park (MD)]: University of Maryland.

[ref55] Su Y. Crain S. (2013, 2013). Children’s knowledge of disjunction and universal quantification in mandarin Chinese. Lang. Linguist. 14, 599–631.

[ref56] Wei H.W. (2018). Adverbial clauses in mandarin Chinese. Qualifying Paper. Los Angeles (CA): University of Southern California.

[ref57] Wei H. W. Li Y. H. A. (2018). “Part 1: preverbal adverbial PPs and clauses” in The syntax of complex sentences in Chinese [special issue]. Linguistic analysis, vol. 42, 163–234.

[ref1009] Winter B. (2013). Linear models and linear mixed effects models in R with linguistic applications. Available at: http://arxiv.org/pdf/1308.5499.pdf (Accessed June 15, 2018).

[ref58] Wu H. Larson R. Liu Y. Liu L. Mar G. (2018). “Rethinking quantifier scope in mandarin” in Proceedings of the north eastern linguistics society annual meeting 48 Vol. 3. eds. Hucklebridge S. Nelson M. (Amherst: University of Massachusetts, GLSA), 257–263.

[ref59] Xu L. Lee T.H.T. (1989). Scope ambiguity and disambiguity in Chinese. Paper presented at the 25th Annual Regional Meeting of the Chicago Linguistic Society.

[ref60] Yang X. Wu Y. (2020). On the scope of quantifier phrases in Chinese passive construction. Int. J. Chin. Linguist. 7, 71–89. doi: 10.1075/ijchl.19010.yan

[ref61] Zhou P. Gao L. (2009). Scope processing in Chinese. J. Psycholinguist. Res. 38, 11–24. doi: 10.1007/s10936-008-9079-x 18521750

[ref62] Zhu D. (1982). *Yufa Jiangyi*.[‘grammar handout’]. Beijing: Commercial Press.

