# Supplementary material for: An experimental investigation into scope rigidity in written Mandarin
Source: Front Psychol. 2023 Jun 9;14:1128616. doi: 10.3389/fpsyg.2023.1128616 (PMC10289302; doi:10.3389/fpsyg.2023.1128616)
Supplement: Supplementary file 1 [file Data_Sheet_1.docx]

Supplementary Material

An experimental investigation into scope rigidity in written Mandarin

# Supplementary Target Sentences

Below is the full list of the target sentences and the contexts associated with them used in the experiment. There are 6 target conditions: *suiran_le, yinwei_le, yaoshi_le, suiran_no le, yinwei_no le, yaoshi_no le*. 36 target context-sentence pairs were created: 6 sets of 6 context-sentence pairs (each set contains one context-sentence pair for each condition). In each set, for the two conditions with the same adverbial type, the context associated with the target sentences is the same and the target sentences are only different on whether the aspect marker *le* is present. To avoid repetition, we list the two conditions with the same adverbial type together: first context then the target sentences for the two conditions. Here we also provide detailed information on all the factors that were balanced for the stimuli, such as the embedded verb type and the type of existential quantificational phrase.

**Set 1**

In this set, the embedded verb is a resultative verb: *chīdiào* ‘eat-up’, and the existential quantificational phrase is in the form of *yī* ‘one/a’ + classifier + noun.

**(1) ‘although’ type adverbial clause:** *suiran_le* condition & *suiran_*no *le* condition

[Context]: 参加最受宠物欢迎的蛋糕师大赛的蛋糕师要做三块不同蛋糕，一共有两条小狗做评委。对于一个蛋糕师来说，如果他做的每块蛋糕都有一条小狗把它吃完，那么他就有可能当选“最佳蛋糕师”称号。蛋糕师大刘做的三块蛋糕的评选结果如下：小狗评委A吃掉了蔬菜蛋糕和胡萝卜蛋糕，小狗评委B吃掉了香蕉蛋糕。大刘满足了入选条件，可是他最后没有得到“最佳蛋糕师”称号，他有点伤心。

[word-for-word glosses of the written context]:

*Cānjiā zuì-shòu-chǒngwù-huānyíng de dàngāoshī dàsài*

attend the.most-enjoy-pet-popularity link pastry.chef competition

*de dàngāoshī yào zuò sān-kuài-bùtóng-dàngāo, yīgòng yǒu*

rel pastry.chef need bake three-clf-different-cake in.total have

*liǎng-tiáo-xiǎogǒu zuò píngwěi. Duìyú yī-ge-dàngāoshī láishuō,*

two- clf-little.dog act.as judge as.for one-clf-pastry.chef as.for

*rúguǒ tā zuò de měi-kuài-dàngāo dōu yǒu yī-tiáo-xiǎogǒu*

if he/she bake rel every-clf-cake all have one-clf-little.dog

*bǎ tā chīwán, nàme tā jiù yǒu kěnéng*

particle it eat.complete so he/she then have possibility

*dāngxuǎn “Zuìjiā dàngāoshī” chēnghào. Dàngāoshī Dàliú zuò de*

be.selected best pastry.chef title pastry.chef Daliu bake rel

*sān-kuài-dàngāo de píngxuǎn jiēguǒ rúxià: xiǎogǒu-píngwěi-A*

three-clf-cake link evaluation result as.follows little.dog-judge-A

*chīdiào-le shūcài-dàngāo hé húluóbo-dàngāo, xiǎogǒu-píngwěi-B* eat.complete-pfv vegetable-cake and carrot-cake little.dog-judge-B

*chīdiào-le xiāngjiāo-dàngāo. Dàliú mǎnzú-le rùxuǎn*

eat.complete-pfv banana-cake Daliu meet--pfv be.selected

*tiáojiàn, kěshì tā zuìhòu méiyǒu dédào “Zuìjiā*

requirement but he in.the.end not receive best

*dàngāoshī” chēnghào, tā yǒudiǎn shāngxīn.*

pastry.chef title he a.bit sad

‘Any pastry chef who participates in the Most Popular Pastry Chef for Pets Contest needs to make three different cakes, and two puppies are judges. For a pastry cook, if every cake he makes has a puppy to eat it, then he may be elected as the “Best Pastry Chef for Pets”. Chef Liu made three cakes and the results were as follows: puppy judge A ate the vegetable cake and carrot cake, and the puppy Judge B ate the banana cake. Chef Liu met the selection criteria, but he did not get the title of “Best Pastry Chef for Pets” in the end. He was a little sad. ‘

[Target sentences]: 虽然一条小狗吃掉**(了)**每块蛋糕，大刘还是没有获得“最佳蛋糕师”称号。

[word-for-word gloss of the target sentences]:

*Suīrán yī-tiáo-xiǎogǒu chīdiào(le) měi-kuài-dàngāo,*

although one-clf-puppy eat-up(-pfv) every-clf-cake

*Dàliú háishi méiyǒu huòdé “Zuìjiā-dàngāoshī” chēnghào.*

Daliu still not receive best-pastry.chef title

‘Although a dog ate/ eats up every cake, Chef Liu still did not get the title of “Best Pastry Chef for Pets”.’

**(2) ‘because’ type adverbial clause:** *yinwei_le* condition & *yinwei_*no *le* condition

[Context]: 最受宠物欢迎的蛋糕师大赛的规则是：参赛的蛋糕师要做三块不同蛋糕，一共有两条小狗做评委；对于一个蛋糕师来说，只要他做的所有蛋糕每一块都有一条小狗把它吃完，他就有可能当选。蛋糕师大刘做的三块蛋糕的评选结果如下：小狗评委A吃掉了蔬菜蛋糕和胡萝卜蛋糕，小狗评委B吃掉了香蕉蛋糕。大刘满足了入选条件，他最后得到了“最佳蛋糕师”称号，他很开心。

[word-for-word glosses of the written context]:

*Zuì-shòu-chǒngwù-huānyíng de dàngāoshī dàsài de guīzé shì:*

the.most-enjoy-pet-popularity link pastry.chef competition link rule is

*cān sài de dàngāoshī yào zuò sān-kuài-bùtóng-dàngāo,*

attend competition rel pastry.chef need bake three-clf-different-cake

*yīgòng yǒu liǎng-tiáo-xiǎogǒu zuò píngwěi; duìyú yī-ge-dàngāoshī*

in.total have two- clf-little.dog act.as judge as.for one-clf-pastry.chef

*láishuō, zhǐyào tā zuò de suǒyǒu-dàngāo měi-yī-kuài*

as.for as.long.as he/she bake rel all-cake every-clf-cake

*dōu yǒu yī-tiáo-xiǎogǒu bǎ tā chīwán, tā jiù yǒu*

all have one-clf-little.dog particle it eat.complete he/she then have

*kěnéng dāngxuǎn. Dàngāoshī dàliú zuò de sān-kuài-dàngāo*

possibility be.selected pastry.chef Daliu bake rel three-clf-cake

*de píngxuǎn jiēguǒ rúxià: xiǎogǒu-píngwěi-A chīdiào-le*

link evaluation result as.follows little.dog-judge-A eat.complete-pfv

*shūcài-dàngāo hé húluóbo-dàngāo, xiǎogǒu-píngwěi-B chīdiào-le*

vegetable-cake and carrot-cake little.dog-judge-B eat.complete-pfv

*xiāngjiāo-dàngāo. Dàliú mǎnzú-le rùxuǎn tiáojiàn, tā zuìhòu*

banana-cake Daliu meet-pfv be.selected requirement he at.the.end

*dédào-le “Zuìjiā-dàngāoshī” chēnghào, tā hěn kāixīn.*

receive-pfv best-pastry.chef title he very happy

‘Any pastry chef who participates in the Most Popular Pastry Chef for Pets Contest needs to make three different cakes, and two puppies are judges. For a pastry cook, if every cake he makes has a puppy to eat it, then he may be elected as the “Best Pastry Chef for Pets”. Chef Liu made three cakes and the results were as follows: puppy judge A ate the vegetable cake and carrot cake, and the puppy judge B ate the banana cake. Chef Liu met the selection criteria, so he got the title of “Best Pastry Chef for Pets” in the end. He was very happy.’

[Target sentences]: 因为一条小狗吃掉**(了)**每块蛋糕，大刘才获得了“最佳蛋糕师”称号。

[word-for-word gloss of the target sentences]:

*Yīnwèi yī-tiáo-xiǎogǒu chīdiào(le) měi-kuài-dàngāo,*

Because one-clf-puppy eat-up(-pfv) every-clf-cake

*DàLiú cái huòdé-le “Zuìjiā-dàngāoshī” chēnghào.*

DaLiu then receive-pfv best-pastry.chef title

‘Because a dog ate/eats up every cake, Chef Liu got the title of “Best Pastry Chef for Pets”.’

**(3) ‘if’ type adverbial clause:** *yaoshi_le* condition & *yaoshi_*no *le* condition

[Context]: 参加最受宠物欢迎的蛋糕师大赛的蛋糕师要做三块不同蛋糕，一共有两条小狗做评委。对于一个蛋糕师来说，如果他做的每块蛋糕都有一条小狗把它吃完，而且每条小狗评委都至少吃掉了他做的一块蛋糕，那么他就有可能当选“最佳蛋糕师”称号。蛋糕师大刘觉得他有希望赢下比赛。

[word-for-word glosses of the written context]:

*Cānjiā zuì-shòu-chǒngwù-huānyíng de dàngāoshī dàsài de*

attend the.most-enjoy-pet-popularity link pastry.chef competition rel

*dàngāoshī yào zuò sān-kuài-bùtóng-dàngāo, yīgòng yǒu*

pastry.chef need bake three-clf-different-cake in.total have

*liǎng-tiáo-xiǎogǒu zuò píngwěi. duìyú yī-ge-dàngāoshī lái-shuō,*

two- clf-little.dog act.as judge as.for one-clf-pastry.chef as.for

*rúguǒ tā zuò de měi-kuài-dàngāo dōu yǒu yī-tiáoxiǎo-gǒu*

if he/she bake rel every-clf-cake all have one-clf-little.dog

*bǎ tā chī-wán, érqiě měi-tiáo-xiǎogǒu píngwěi dōu zhìshǎo*

particle it eat.complete moreover every-clf-little.dog judge all at.least

*chīdiào-le tā zuò de yī-kuài-dàngāo, nàme tā jiù yǒu*

eat.complete-pfv he/she bake rel one-clf-cake so he/she then have

*kěnéng dāngxuǎn “Zuìjiā-dàngāoshī” chēnghào. Dàngāoshī dàliú*

possibility be.selected best-pastry.chef title pastry.chef Daliu

*juéde tā yǒu xīwàng yíng-xià bǐsài.*

think he have hope win competition

‘Any pastry chef who participates in the Most Popular Pastry Chef for Pets Competition needs to make three different cakes, and two puppies are judges. For a pastry cook, if every cake he makes has a puppy to eat it and every puppy judge eats up at least one of the cakes he makes, then he may be elected as the “Best Pastry Chef for Pets”. Chef Liu is very confident that he could win the competition.’

[Target sentences]: 要是一条小狗吃掉**(了)**每块蛋糕，大刘就有希望获得“最佳蛋糕师”称号。

[word-for-word gloss of the target sentences]:

*Yàoshi yī-tiáo-xiǎogǒu chīdiào(le) měi-kuài-dàngāo,*

if one-clf-puppy eat-up(-pfv) every-clf-cake

*DàLiú jiù yǒu xīwàng huòdé “Zuìjiā-dàngāoshī” chēnghào.*

DaLiu then have hope receive best-pastry.chef title

‘If a dog ate/eats up every cake, Chef Liu then is likely to get the title of “Best Pastry Chef for Pets”.’

**Set 2**

In this set, the embedded verb is a resultative verb: *dá-duì* ‘answer-correct’, and the existential quantificational phrase is in the form of *liǎng* ‘two’ + classifier + noun.

**(4) ‘although’ type adverbial clause:** *suiran_le* condition & *suiran_*no *le* condition

[Context]: 奥数集训队选拔赛规则是：参赛队每队三个人，一共三道题；对于一个参赛队来说，三道题中每一道题都派两名队员出来答题，每道题都答对，这个队就有可能获得入选资格。海淀队的队员小王、小蒋和小郑的答题情况如下：小王和小蒋答对了前两道题，小蒋和小郑答对了最后一道题。海淀队满足了入选条件，可是最后没能入选集训队。小王、小蒋和小郑很伤心。

[word-for-word glosses of the written context]:

*Àoshǔ jíxùn-duì xuǎnbá-sài guīzé shì: cānsài-duì*

Math.Olympiad training.team qualification-exam rule is attend.contest-team

*měi-duì sān-ge-rén, yīgòng sān-dào-tí; duìyú*

every-team three-clf-person in.total three-clf-problem.set as.for

*yī-ge-cānsài-duì láishuō, sān-dào-tí zhōng*

one-clf-attend.contest-team as.for three-clf-problem.set among

*měi-yī-dào-tí dōu pài liǎng-míng-duìyuán chūlái*

every-clf- problem.set all send two-clf-team.member come.out

*dātí, měi-dào-tí dōu dáduì, zhè-ge-duì jiù*

answer.question every-clf-problem.set all answer.correct this-clf-team then

*yǒu kěnéng huòdé rùxuǎn zīgé. Hǎidiàn-duì de duìyuán*

have possibility receive be.selected qualification Haidian-team link team.member

*Xiǎowáng, Xiǎojiǎng hé Xiǎozhèng de dātí qíngkuàng rúxià:*

Xiaowang, Xiaojiang and Xiaozheng link answer.question result as.follows

*Xiǎowáng hé Xiǎojiǎng dáduì-le qián-liǎng-dào-tí, Xiǎojiǎng*

Xiaowang and Xiaojiang answer.correct-pfv first-two-clf-problem.set Xiaojiang

*hé Xiǎozhèng dáduì-le zuìhòu-yī-dào-tí. Hǎidiàn- duì*

and Xiaozheng answer.correct-pfv last-two-clf-problem.set Haidian-team

*mǎnzú-le rùxuǎn tiáojiàn, kěshì zuìhòu méi néng rùxuǎn*

meet-pfv be.selected requirement but in.the.edn not able be.selected

*jíxùn-duì. Xiǎowáng, Xiǎojiǎng hé Xiǎozhèng hěn shāngxīn*

training-team. Xiaowang, Xiaojiang and Xiaozheng very sad

‘The rules of the qualification exam for the Math Olympiad training team are: each team consists of three members and there are three problem sets in total; for a team, each of the three problem set needs to be answered by two members of that team and a team is possible to join the Math Olympic training program when that team gives correct answers for all three problem sets. Three members of the Haidian team are Xiao Wang, Xiao Jiang and Xiao Zheng. Xiao Wang and Xiao Jiang answered the first two problem sets correctly. Xiao Jiang and Xiao Zheng answered the last problem set correctly. The Haidian team met the selection criteria, but they were not selected to join the training program. Xiao Wang, Xiao Jiang and Xiao Zheng were very sad.’

[Target sentences]: 虽然两个学生答对**(了)**每道题，海淀队还是没有入选奥数集训队。

[word-for-word gloss of the target sentences]:

*Suīrán liǎng-gè-xuésheng dá-duì(-le) měi-dào-tí,*

although two-clf-student answer-correct(-pfv) every-clf-problem.set

*Hǎidiàn-duì háishi méiyǒu rùxuǎn àoshǔ jíxùn-duì.*

Haidian-team still not be.selected Math.Olympiad training-team

‘Although two students answer(ed) every problem set correctly, Haidian team still did not get selected to join the Math Olympiad training program.’

**(5) ‘because’ type adverbial clause:** *yinwei_le* condition & *yinwei*_no *le* condition

[Context]: 奥数集训队选拔赛规则是：参赛队每队三个人，一共三道题；对于一个参赛队来说，三道题中每一道题都派两名队员出来答题，每道题都答对，这个队就有可能获得入选资格。海淀队的队员小王、小蒋和小郑的答题情况如下：小王和小蒋答对了前两道题，小蒋和小郑答对了最后一道题。海淀队满足了入选条件，小王、小蒋和小郑很高兴。

[word-for-word glosses of the written context]:

*Àoshǔ jíxùn-duì xuǎnbá-sài guīzé shì: cānsài-duì*

Math.Olympiad training.team qualification-exam rule is attend.contest-team

*měi-duì sān-ge-rén, yīgòng sān-dào-tí; duìyú*

every-team three-clf-person in.total three-clf-problem.set as.for

*yī-ge-cānsài-duì láishuō, sān-dào-tí zhōng*

one-clf-attend.contest-team as.for three-clf-problem.set among

*měi-yī-dào-tí dōu pài liǎng-míng-duìyuán chūlái*

every-clf- problem.set all send two-clf-team.member come.out

*dātí, měi-dào-tí dōu dáduì, zhè-ge-duì jiù*

answer.question every-clf-problem.set all answer.correct this-clf-team then

*yǒu kěnéng huòdé rùxuǎn zīgé. Hǎidiàn-duì de duìyuán*

have possibility receive be.selected qualification Haidian-team link team.member

*Xiǎowáng, Xiǎojiǎng hé Xiǎozhèng de dātí qíngkuàng rúxià:*

Xiaowang, Xiaojiang and Xiaozheng link answer.question result as.follows

*Xiǎowáng hé Xiǎojiǎng dáduì-le qián-liǎng-dào-tí, Xiǎojiǎng*

Xiaowang and Xiaojiang answer.correct-pfv first-two-clf-problem.set Xiaojiang

*hé Xiǎozhèng dáduì-le zuìhòu-yī-dào-tí. Hǎidiàn- duì*

and Xiaozheng answer.correct-pfv last-two-clf-problem.set Haidian-team

*mǎnzú-le rùxuǎn tiáojiàn, Xiǎowáng, Xiǎojiǎng hé Xiǎozhèng*

meet-pfv be.selected requirement Xiaowang, Xiaojiang and Xiaozheng

*hěn gāoxìng.*

very happy

‘The rules of the qualification exam for the Math Olympiad training team are: each team consists of three members and there are three problem sets in total; for a team, each of the three problem set needs to be answered by two members of that team and a team is possible to join the Math Olympic training program when that team gives correct answers for all three problem sets. Three members of the Haidian team are Xiao Wang, Xiao Jiang and Xiao Zheng. Xiao Wang and Xiao Jiang answered the first two problem sets correctly. Xiao Jiang and Xiao Zheng answered the last problem set correctly. The Haidian team met the selection criteria, and they were selected to join the training program. Xiao Wang, Xiao Jiang and Xiao Zheng were very happy.’

[Target sentences]: 因为两个学生答对**(了)**每道题，海淀队才入选了奥数集训队。

[word-for-word gloss of the target sentences]:

*Yīnwèi liǎng-gè-xuésheng dá-duì(le) měi-dào-tí,*

because two-clf-student answer-correct(-pfv) every-clf-problem.set

*Hǎidiàn-duì cái rùxuǎn-le àoshǔ jíxùn-duì.*

Haidian-team only.then be.selected-pfv Math.Olympiad training.team

‘Because two students answer(ed) every problem set correctly, Haidian team was selected to join the Math Olympiad training program.’

**(6) ‘if’ type adverbial clause:** *yaoshi*_*le* condition & *yaoshi*_no *le* condition

[Context]: 参加奥数集训队选拔赛的各个代表队每队由三个学生组成，一共要回答三道题，每次答题每个队伍要派出两名队员来回答，而且每个成员都要参加至少一个比赛环节。对于一个参赛队来说，如果这个队伍每次去答题的两个学生都能给出题目的正确答案，那么这个队就有可能获得入选资格。海淀队的队员觉得他们有希望赢下比赛。

[word-for-word glosses of the written context]:

*Cānjiā àoshǔ jíxùn-duì xuǎnbá-sài de gègè*

attend.competition math.Olympid training-team qualification-exam rel every

*dàibiǎo-duì měi-duì yóu sān-ge-xuésheng zǔchéng,*

representative-team every-team by three-clf-student is.consisted

*yīgòng yào huídá sān-dào-tí, měi-cì dā-tí*

in.total need answer three-clf-problem.set every-time answer-question

*měi-gè-duìwǔ yào pàichū liǎng-míng-duìyuán lái*

every-clf-team need send two-clf-team.member come

*huídā, érqiě měi-gè-chéngyuán dōu yào*

answer moreover every-clf-team.member all need

*cānjiā zhìshǎo yī-ge-bǐsài-huánjié. duìyú*

attend at.least every-clf-competition-session as.for

*yī-ge-cānsài-duì láishuō, rúguǒ zhè-ge-duìwǔ měi-cì qù*

one-clf-attend.competition-team as.for if this-clf-team every-time go

*dātí de liǎng-gè-xuésheng dōu néng gěichū tímù de*

answer.question rel two-clf-student all can offer question link

*zhèngquè dá'àn, nàme zhè-ge-duì jiù yǒu kěnéng*

correct answer so this-clf-team then have possibility

*huòdé rùxuǎn zīgé. Hǎidiàn-duì de duìyuán*

receive be.selected qualification Haidian-team link team.member

*juéde tāmen yǒu xīwàng yíngxià bǐsài.*

think they have hope win competition

‘The rules of the qualification exam for the Math Olympiad training team are: each team consists of three members and there are three problem sets in total; for a team, each of the three problem set needs to be answered by two members of that team and every member must represent that team at least once. A team is possible to join the Math Olympic training program if that team gives correct answers for all three problem sets. Three members of the Haidian team are very confident that they can be selected to join the training program.’

[Target sentence for *yaoshi_le* condition]: 要是两个学生答对**(了)**每道题，海淀队就有希望入选奥数集训队。

[word-for-word gloss of the target sentences]:

*Yàoshi liǎng-gè-xuésheng dá-duì(le) měi-dào-tí,*

if two-clf-student answer-correct(-pfv) every-clf-problem.set

*Hǎidiàn-duì jiù yǒu xīwàng rùxuǎn ào shǔ jíxùn-duì.*

Haidian-team then have hope get.selected Math.Olympiad training.program

‘If two students answer(ed) every problem set correctly, Haidian team then is likely to join the Math Olympiad training program.’

**Set 3**

In this set, the embedded verb is a resultative verb: *dā-chū* ‘build-up’, and the existential quantificational phrase is in the form of *sān* ‘three’ + classifier + noun.

**(7) ‘although’ type adverbial clause:** *suiran_le* condition & *suiran_*no *le* condition

[Context]: 乐高积木搭房子比赛的规则是，每个参赛队成员应在4-6人之间，一共有三个搭房子的比赛环节，每个队伍每个比赛环节要派出3名队员参赛。对一个参赛队来说，如果这个队伍在每个环节都搭出了规定样式的房子，那这个队伍就算是闯关成功；但是，只有耗时最短的队伍才能获得一万美金的奖金。小泽队的队员小王、小蒋和小泽搭出了前两个积木房子，小泽、小敏和小林搭出了最后一个积木房子。小泽队闯关成功了，但是最后因为用时太长，没能拿到奖金。

[word-for-word glosses of the written context]:

*Lègāo jīmù dā-fángzi bǐsài de guīzé*

Lego building.blocks build-buildings competition link rule

*shì, měi-gè-cānsài-duì-chéngyuán yìng zài 4-6 rén*

is every-clf-attend.competition-team-members should be 4-6 person

*zhījiān, yīgòng yǒu sān-ge-dā-fángzi de bǐsài huánjié,*

within in.total have three-clf-build-buldings link competition session

*měi-gè-duìwǔ měi-gè-bǐsài-huánjié yào pàichū*

every-clf-team every-clf-attend.competition-session need send

*3-míng-duìyuán cānsài. duì yī-ge-cānsài-duì*

three-clf-member attend.competition as.for one-clf-attend.competition-team

*láishuō, rúguǒ zhè-ge-duìwǔ zài měi-gè-huánjié dōu dā-chū-le*

as.for if this-clf-team at every-clf-session all build-up-pfv

*guīdìng yàngshì de fángzi, nà zhè-ge-duìwǔ jiù suàn*

set format link building so this-clf-team then count.as

*shì chuǎngguān chénggōng; dànshì, zhǐyǒu hàoshí zuì duǎn*

be pass.through successfully but only consume.time most short

*de duìwǔ cái néng huòdé yīwàn-měijīn de jiǎngjīn.*

rel team only can be.awarded ten.thousand-dollars link award.money

*Xiǎozé-duì de duìyuán Xiǎowáng, Xiǎojiǎng hé Xiǎozé*

Xiaoze-team link member Xiaowang Xiaojiang and Xiaoze

*dā-chū-le qián-liǎng-gè-jīmù-fángzi, Xiǎozé, Xiǎomǐn hé Xiǎolín*

build-up-pfv first-two-clf-building.blocks-buildings Xiaoze Xiaomin and Xiaolin

*dā-chū-le zuìhòu-yī-ge-jīmù-fángzi. Xiǎozé-duì chuǎngguān*

build-up-pfv last-one-clf-building.blocks-buildings Xiaoze-team pass.through

*chénggōng-le, dànshì zuìhòu yīnwèi yòngshí tài cháng,*

successfully-pfv but in.the.end because consume.time too long

*méi néng nádào jiǎngjīn.*

not able receive award.money

‘To attend the Lego House Building Team Competition, each team should have 4-6 team members. The competition includes three rounds. For each round, each team needs to send 3 players to attend. A team can be considered as a finalist if the team successfully builds up a house as required at each round; but only the team that spends the shortest time can win a prize of 10,000 dollars. Xiaoze Team has 5 members. Xiao Wang, Xiao Jiang and Xiao Ze successfully finished the first two rounds, and Xiao Ze, Xiao Min and Xiao Lin finished the third round. The Xiaoze Team was considered as a finalist, but in the end this team didn’t get the prize because they took too long to finish the three rounds.’

[Target sentences]: 虽然三个人搭出**(了)**每个积木房子，小泽队还是没能拿到一万美金奖金。

[word-for-word gloss of the target sentences]:

*Suīrán sān-ge-rén dā-chū(-le) měi-gè-jīmù-fángzi,*

although three-clf-person build-up(-pfv) every-clf-building.blocks-buildings

*Xiǎozé-duì háishi méi néng nádào yīwàn-měijīn-jiǎngjīn.*

Xiaoze-team still not able.to win ten.thousand-dollar-award.money

‘Although three persons build/built up every Lego building, Xiaoze team still did not win the prize of 10,000 US dollars.’

**(8) ‘because’ type adverbial clause:** *yinwei_le* condition & *yinwei_*no *le* condition

[Context]: 乐高积木搭房子比赛的规则是，每个参赛队成员应在4-6人之间，一共有三个搭房子的比赛环节，每个队伍每个比赛环节要派出3名队员参赛。对一个参赛队来说，如果这个队伍在每个环节都搭出了规定样式的房子，那这个队伍就可以入选最佳乐高团队。小泽队的队员小王、小蒋和小泽搭出了前两个积木房子，小泽、小敏和小林搭出了最后一个积木房子。最后小泽队获选最佳乐高团队。

[word-for-word glosses of the written context]:

*Lègāo jīmù dā-fángzi bǐsài de guīzé*

Lego building.blocks build-buildings competition link rule

*shì, měi-gè-cānsài-duì-chéngyuán yìng zài 4-6 rén*

is every-clf-attend.competition-team-members should be 4-6 person

*zhījiān, yīgòng yǒu sān-ge-dā-fángzi de bǐsài huánjié,*

within in.total have three-clf-build-buldings link competition session

*měi-gè-duìwǔ měi-gè-bǐsài-huánjié yào pàichū*

every-clf-team every-clf-attend.competition-session need send

*3-míng-duìyuán cānsài. duì yī-ge-cānsài-duì*

three-clf-member attend.competition as.for one-clf-attend.competition-team

*láishuō, rúguǒ zhè-ge-duìwǔ zài měi-gè-huánjié dōu dā-chū-le*

as.for if this-clf-team at every-clf-session all build-up-pfv

*guīdìng yàngshì de fángzi, nà zhè-ge-duìwǔ jiù*

set format link building so this-clf-team then

*kěyǐ rùxuǎn zuìjiā-Lègāo-tuánduì. Xiǎozé-duì de duìyuán Xiǎowáng*,

can be.selected best-Lego-team Xiaoze-team link member Xiaowang

*Xiǎojiǎng hé Xiǎozé dā-chū-le qián-liǎng-gè-jīmù-fángzi,*

Xiaojiang and Xiaoze build-up-pfv first-two-clf-building.blocks-buildings

*Xiǎozé, Xiǎomǐn hé Xiǎolín dā-chū-le zuìhòu-yī-ge-jīmù-fángzi.*

Xiaoze Xiaomin and Xiaolin build-up-pfv last-one-clf-building.blocks-buildings

*zuìhòu Xiǎozé-duì huòxuǎn zuìjiā-Lègāo-tuánduì.*

in.the.end Xiaoze-team be.selected best-Lego-team

‘To attend the Lego House Building Team Competition, each team should have 4-6 team members. The competition includes three rounds. For each round, each team needs to send 3 players to attend. A team can be considered as “Best Lego Team” if the team successfully builds up a house as required at each round; but only the team that spends the shortest time can win a prize of 10,000 dollars. Xiaoze Team has 5 members. Xiao Wang, Xiao Jiang and Xiao Ze successfully finished the first two rounds, and Xiao Ze, Xiao Min and Xiao Lin finished the third round. The Xiaoze Team was selected as the “Best Lego Team” in the end.’

[Target sentences]: 因为三个人搭出**(了)**每个积木房子，小泽队入选了最佳乐高团队。

[word-for-word gloss of the target sentences]:

*Yīnwèi sān-ge-rén dā-chū(-le) měi-gè-jīmù-fángzi,*

because three-clf-person build-up(-pfv) every-clf-building.blocks-buildings *Xiǎozé-duì rùxuǎn-le zuìjiā-Lègāo-tuánduì.*

Xiaoze-team get.selected-pfv best-Lego-team

‘Because three persons build/built up every Lego building, Xiaoze team was selected as the “Best Lego Team”.’

**(9) ‘if’ type adverbial clause:** *yaoshi_le* condition & *yaoshi_*no *le* condition

[Context]: 乐高积木搭房子比赛的规则是，每个参赛队成员应在4-6人之间，一共有三个搭房子的比赛环节，每个队伍每个比赛环节要派出3名队员参赛，而且每个成员都要参加至少一个比赛环节。对一个参赛队来说，如果这个队伍在每个环节都搭出了规定样式的房子，那这个队伍就算是闯关成功。小泽队的队员对这场比赛信心十足。

[word-for-word glosses of the written context]:

*Lègāo jīmù dā-fángzi bǐsài de guīzé*

Lego building.blocks build-buildings competition link rule

*shì, měi-gè-cānsài-duì-chéngyuán yìng zài 4-6 rén*

is every-clf-attend.competition-team-members should be 4-6 person

*zhījiān, yīgòng yǒu sān-ge-dā-fángzi de bǐsài huánjié,*

within in.total have three-clf-build-buldings link competition session

*měi-gè-duìwǔ měi-gè-bǐsài-huánjié yào pàichū*

every-clf-team every-clf-attend.competition-session need send

*3-míng-duìyuán cānsài, érqiě měi-gè-chéngyuán dōu yào*

three-clf-member attend.competition moreover every-clf-member all need

*cānjiā zhìshǎo yī-ge-bǐsài-huánjié. duì yī-ge-cānsài-duì*

attend at.least one-clf-competition-session as.for one-clf-attend.competition-team

*láishuō, rúguǒ zhè-ge-duìwǔ zài měi-gè-huánjié dōu dā-chū-le*

as.for if this-clf-team at every-clf-session all build-up-pfv

*guīdìng yàngshì de fángzi, nà zhè-ge-duìwǔ jiù suàn*

set format link building so this-clf-team then count.as

*shì chuǎngguān chénggōng. Xiǎozé-duì de duìyuán duì*

be pass.through successfully Xiaoze-tem link member toward

*zhè-cháng-bǐsài xìnxīn shízú.*

this-clf-competition confidence full.of

‘To attend the Lego House Building Team Competition, each team should have 4-6 team members. The competition includes three rounds. For each round, each team needs to send 3 players to attend. A team can be considered as a finalist if the team successfully builds up a house as required at each round. All the team members of Xiaoze Team are very confident that their team can be a finalist.’

[Target sentences]: 要是三个人搭出**(了)**每个积木房子，小泽队就算闯关成功。

[word-for-word gloss of the target sentences]:

*Yàoshi sān-ge-rén dā-chū(-le) měi-gè-jīmù-fángzi,*

If three-clf-person build-up(-pfv) every-clf-building.blocks-buildings

*Xiǎozé-duì jiù suàn chuǎngguān chénggōng.*

Xiaoze-team then count.as pass.through successfully

‘If three persons build/built up every Lego building, Xiaoze team then would be considered as a finalist.’

**Set 4**

In this set, the embedded verb is a durative verb: *xiézhù* ‘assist’, and the existential quantificational phrase is in the form of *yī* ‘one/a’ + classifier + noun.

**(10) ‘although’ type adverbial clause:** *suiran_le* condition & *suiran_*no *le* condition

[Context]: 往届科技博览会，常常是一个志愿者负责协助三个展台的布置、咨询引导等事务，展览会结束后，参展方常常抱怨志愿者人手不够。今年这届博览会，主办方招募到了很多志愿者，人手充足，给每个展台安排了一个志愿者去协助。可是今年观展人数也大幅度上升了，最后参展方还是抱怨志愿者人手不够。

[word-for-word glosses of the written context]:

*Wǎng-jiè-kējì-bólǎnhuì, chángcháng shì yī-ge-zhìyuànzhě fùzé*

previous-clf-technology-exhibition often foc one-clf-volunteer in.charge.of

*xiézhù sān-ge-zhǎntái de bùzhì, zīxún yǐndǎo*

assist three-clf-booth link arrangement consultation guidance

*děng shìwù, zhǎnlǎnhuì jiéshù hòu, cānzhǎnfāng chángcháng*

and.so.on matters exhibition finish after vendor often

*bàoyuàn zhìyuànzhě rénshǒu bù gòu. jīnnián zhè-jiè-bólǎnhuì,*

complain volunteer staffing not adequate this.year this-clf-exhibition

*zhǔbànfāng zhāomù-dào-le hěnduō zhìyuànzhě, rénshǒu chōngzú,*

organizer recruit-complete-pfv many volunteer staffing adequate

*gěi měi-gè-zhǎntái ānpái-le yī-gè-zhìyuànzhě qù xiézhù.*

for every-clf-booth allocate-pfv one-clf-volunteer go assist

*kěshì jīnnián guān zhǎn rénshù yě dàfúdù*

but this.year visit exhibition head.count also significantly

*shàngshēng-le, zuìhòu cānzhǎnfāng háishi bàoyuàn zhìyuànzhě*

increase-pfv in.the.end vendor still complain volunteer

*rénshǒu bù gòu.*

staffing not adequate

‘In the past, one single volunteer at the Science and Technology Exhibition needed to help vendors with the arrangement, consultation and guidance of the three booths. After the exhibition, the exhibitors often complained that they were short of hands and there were not enough volunteers to help them. At this year’s exhibition, the organizers recruited a lot of volunteers and had adequate. A volunteer was arranged for each booth to assist. However, the number of visitors has also increased significantly this year. In the end, it turned out that the vendors still complained about being shorthanded.’

[Target sentences]: 虽然一个志愿者协助**(了)**每个展台，这届科技博览会的参展方还是抱怨志愿者人手不够。

[word-for-word gloss of the target sentences]:

*Suīrán yī-ge-zhìyuànzhě xiézhù(-le) měi-gè-zhǎntái, zhè-jiè-kējì-*

Although one-clf-volunteer assist(-pfv) every-clf-booth this-clf-technology

*bólǎnhuì de cānzhǎnfāng háishi bàoyuàn zhìyuànzhě rénshǒu*

exhibition link vendor still complain volunteer staffing

*bù gòu.*

not adequate

‘Although a volunteer assist(ed) every booth, the vendors at the exhibition of this year still complained about being shorthanded.’

**(11) ‘because’ type adverbial clause:** *yinwei_le* condition & *yinwei_*no *le* condition

[Context]: 往届科技博览会，常常是一个志愿者负责协助三个展台的布置、咨询引导等事务，展览会结束后，参展方常常抱怨志愿者人手不够，建议给每个展台配置一个志愿者。今年这届博览会，主办方招募到了很多志愿者，人手充足，给每个展台安排了一个志愿者去协助。参展方今年表示很满意。

[word-for-word glosses of the written context]:

*Wǎng-jiè-kējì-bólǎnhuì, chángcháng shì yī-gè-zhìyuànzhě fùzé*

previous-clf-technology-exhibition often foc one-clf-volunteer in.charge.of

*xiézhù sān-ge-zhǎntái de bùzhì, zīxún yǐndǎo*

assist three-clf-booth link arrangement consultation guidance

*děng shìwù, zhǎnlǎnhuì jiéshù hòu, cānzhǎnfāng chángcháng*

and.so.on matters exhibition finish after vendor often

*bàoyuàn zhìyuànzhě rénshǒu bù gòu. jiànyì gěi měi-gè-zhǎntái*

complain volunteer staffing not adequate suggest for every-clf-booth

*pèizhì yī-gè-zhìyuànzhě, jīnnián zhè-jiè-bólǎnhuì, zhǔbànfāng*

allocate one-clf-volunteer this.year this-clf-exhibition organizer

*zhāomù-dào-le hěnduō zhìyuànzhě, rénshǒu chōngzú,*

recruit-complete-pfv many volunteer staffing adequate

*gěi měi-gè-zhǎntái ānpái-le yī-gè-zhìyuànzhě qù xiézhù.*

for every-clf-booth allocate-pfv one-clf-volunteer go assist

*cānzhǎnfāng jīnnián biǎoshì hěn mǎnyì.*

vendor this.year express very satisfactory

‘In the past, one single volunteer at the Science and Technology Exhibition needed to help vendors with the arrangement, consultation and guidance of the three booths. After the exhibition, the exhibitors often complained that they were short of hands and there were not enough volunteers to help them. At this year’s exhibition, the organizers recruited a lot of volunteers and had sufficient staff. A volunteer was arranged for each booth to assist. In the end, it turned out that the exhibitors were very satisfactory with this arrangement.’

[Target sentences]: 因为一个志愿者协助**(了)**每个展台，这届科技博览会的参展方没有抱怨志愿者人手不够。

[word-for-word gloss of the target sentences]:

*Yīnwèi yī-ge-zhìyuànzhě xiézhù(-le) měi-gè-zhǎntái, zhè-jiè-kējì*

because one-clf-volunteer assist(-pfv) every-clf-booth this-clf-technology

*bólǎnhuì de cānzhǎnfāng méiyǒu bàoyuàn zhìyuànzhě rénshǒu*

exhibition link vendor not complain volunteer staffing

*bù gòu.*

not adequate

‘Because a volunteer assist(ed) every booth, the vendors at the exhibition of this year did not complain about being shorthanded.’

**(12) ‘if’ type adverbial clause:** *yaoshi_le* condition & *yaoshi*_no *le* condition

[Context]: 往届科技博览会，常常是一个志愿者负责协助三个展台的布置、咨询引导等事务；展览会结束后，参展方常常抱怨志愿者人手不够，建议给每个展台配置一个志愿者。这届科技博览会组委会打算给每个展台安排一个志愿者，这样参展方就不会抱怨了。

[word-for-word glosses of the written context]:

*Wǎng-jiè-kējì-bólǎnhuì, chángcháng shì yī-gè-zhìyuànzhě fùzé*

previous-clf-technology-exhibition often foc one-clf-volunteer in.charge.of

*xiézhù sān-ge-zhǎntái de bùzhì, zīxún yǐndǎo*

assist three-clf-booth link arrangement consultation guidance

*děng shìwù, zhǎnlǎnhuì jiéshù hòu, cānzhǎnfāng chángcháng*

and.so.on matters exhibition finish after vendor often

*bàoyuàn zhìyuànzhě rénshǒu bù gòu. jiànyì gěi měi-gè-zhǎntái*

complain volunteer staffing not adequate suggest for every-clf-booth

*pèizhì yī-gè-zhìyuànzhě. zhè-jiè-bólǎnhuì, zǔwěihuì*

allocate one-clf-volunteer this-clf- technology-exhibition organizer

*dǎsuàn gěi měi-gè-zhǎntái ānpái yī-ge-zhìyuànzhě, zhèyàng*

plan for every-clf-booth allocate one-clf-volunteer this.way

*cānzhǎnfāng jiù bù huì bàoyuàn-le.*

vendor then not will complain-pfv

‘In the past, one single volunteer at the Science and Technology Exhibition needed to help vendors with the arrangement, consultation and guidance of the three booths. After the exhibition, the exhibitors often complained that they were short of hands and there were not enough volunteers to help them. The exhibitors suggested to the organizers that there should one volunteer for each booth. The organizers plan to do so for this year’s exhibition so that vendors would not complain about being shorthanded.’

[Target sentences]: 要是一个志愿者协助**(了)**每个展台，这届科技博览会的参展方就不会抱怨志愿者人手不够了。

[word-for-word gloss of the target sentences]:

*Yàoshi yī-ge-zhìyuànzhě xiézhù(-le) měi-gè-zhǎntái, zhè-jiè-kējì-*

if one-clf-volunteer assist(-pfv) every-clf-booth this-clf-technology

*bólǎnhuì de cānzhǎnfāng jiù bù huì bàoyuàn zhìyuànzhě*

exhibition link vendor then not will complain volunteer

*rénshǒu bù gòu-le.*

staffing not adequate-pfv

‘If a volunteer assist(ed) every booth, the vendors at the exhibition of this year would not complain about being shorthanded anymore.’

**Set 5**

In this set, the embedded verb is a durative verb: *bùzhì* ‘decorate (a room)’, and the existential quantificational phrase is in the form of *liǎng* ‘two’ + classifier + noun.

**(13) ‘although’ type adverbial clause:** *suiran_le* condition & *suiran_*no *le* condition

[Context]: 振华学校的传统是，每月月末由一个班级负责全校所有教室的布置任务。以往教室布置的任务，都是一个学生负责一间教室，教室布置任务常常也是敷衍了事。这个月轮到了三班负责全校的教室布置。三班主任王老师给每间教室派去了两名学生布置，让两个学生互相监督、分工合作。可是协调沟通不畅，这次教室布置还是出现了很大的纰漏。

[word-for-word glosses of the written context]:

*Zhènhuá-xuéxiào de chuántǒng shì, měi-yuè-yuèmò yóu*

Zhenhua-school link tradition is every-month-end.of.month by

*yī-ge-bānjí fùzé quán-jiào suǒyǒu jiàoshì de bùzhì*

one-clf-class responsible all-school all classroom link decoration

*rènwu. yǐwǎng jiàoshì bùzhì de rènwu, dōu shì*

task in.the.past classroom decoration link task all foc

*yī-ge-xuésheng fùzé yī-jiān-jiàoshì, jiàoshì bùzhì*

one-clf-student responsible one-clf-classroom classroom decoration

*rènwu chángcháng yě shì fūyǎnliǎoshì. Zhè-ge-yuè*

task often also foc perfunctorily this-clf-month

*lúndào-le sān-bān fùzé quán-jiào de jiàoshì bùzhì.*

be.up.to-pfv Class.Three responsible all-school link classroom decoration

*sānbān-zhǔrèn-wánglǎoshī gěi měi-jiān-jiàoshì pàiqù-le*

Class.Three-mentor-Teacher.Wang for every-clf-classroom send-pfv

*liǎng-míng-xuésheng bùzhì, ràng liǎng-gè-xuésheng hùxiāng*

two-clf-student decoration let two-clf-student each.other

*jiāndū, fēngōng hézuò. kěshì xiétiáo gōutōng*

supervise share.work collaborate but coordination communication

*bù chàng, zhè-cì-jiàoshì-bùzhì háishi chūxiàn-le.*

not smooth this-clf-classroom-decoration still happen-pfv

*hěn dà de pīlòu.*

very big link mistake

‘At Zhenhua High School, one class is responsible for the decorations of all classrooms at the end of each month. In the past, a class often sent one student to decorate each classroom, and students often did not take the decoration task seriously. For this month, it is the turn of the Class Three which is responsible for the decorations of all classrooms. The mentor of the Class Three, Mr. Wang, decided to send two students to each classroom, so that these two students can work together and collaboratively complete the decoration task. However, due to lack of coordination and communication, the decoration task was not completed successfully and smoothly.’

[Target sentences]: 虽然两名学生布置**(了)**每间教室，这次教室布置还是出现了很大的纰漏。

[word-for-word gloss of the target sentences]:

*Suīrán liǎng-míng-xuésheng bùzhì(-le) měi-jiān-jiàoshì,*

although two-clf-student decorate(-pfv) every-clf-classroom

*zhè-cì-jiàoshì-bùzhì háishi chūxiàn-le hěn dà de pīlòu.*

this-clf-classroom-decoration still happen-pfv very big link mistake

‘Although two students decorate/decorated every classroom, the decoration task still was not completed successfully and smoothly this time.’

**(14) ‘because’ type adverbial clause:** *yinwei_le* condition & *yinwei_*no *le* condition

[Context]: 振华学校的传统是，每月月末由一个班级负责全校所有教室的布置任务。以往教室布置的任务，都是一个学生负责两间教室，教室布置任务常常也是敷衍了事。这个月轮到了三班负责全校的教室布置。三班主任王老师给每间教室派去了两名学生布置，两个人分工合作，完成得又快又好。这次教室布置效果非常好，得到了全校师生的称赞。

[word-for-word glosses of the written context]:

*Zhènhuá-xuéxiào de chuántǒng shì, měi-yuè-yuèmò yóu*

Zhenhua-school link tradition is every-month-end.of.month by

*yī-ge-bānjí fùzé quán-jiào suǒyǒu jiàoshì de bùzhì*

one-clf-class responsible all-school all classroom link decoration

*rènwu. yǐwǎng jiàoshì bùzhì de rènwu, dōu shì*

task in.the.past classroom decoration link task all foc

*yī-ge-xuésheng fùzé yī-jiān-jiàoshì, jiàoshì bùzhì*

one-clf-student responsible one-clf-classroom classroom decoration

*rènwu chángcháng yě shì fūyǎnliǎoshì. Zhè-ge-yuè*

task often also foc perfunctorily this-clf-month

*lúndào-le sān-bān fùzé quán-jiào de jiàoshì bùzhì.*

be.up.to-pfv Class.Three responsible all-school link classroom decoration

*sānbān-zhǔrèn-wánglǎoshī gěi měi-jiān-jiàoshì pàiqù-le*

Class.Three-mentor-Teacher.Wang for every-clf-classroom send-pfv

*liǎng-míng-xuésheng bùzhì, liǎng-gè-rén fēngōng hézuò,*

two-clf-student decoration two-clf-person share.work collaborate

*wánchéng de yòu-kuài-yòu-hǎo. Zhè-cì-jiàoshì-bùzhì xiàoguǒ*

finish link both-quick-and-good this-time-classroom-decoration result

*fēicháng hǎo, dédào-le quán-xiào shīshēng de chēngzàn.*

very good receive-pfv all-scholl faculty.student link praise

‘At Zhenhua High School, one class is responsible for the decorations of all classrooms at the end of each month. In the past, a class often sent one student to decorate each classroom, and students often did not take the decoration task seriously. For this month, it is the turn of the Class Three which is responsible for the decorations of all classrooms. The mentor of the Class Three, Mr. Wang, decided to send two students to each classroom. For each classroom, there were two students working together and they collaboratively completed the decoration task with efficiency. All the faculty and students in the school spoke highly of the final results.

[Target sentences]: 因为两名学生布置**(了)**每间教室，这次教室布置没有出现很大的纰漏。

[word-for-word gloss of the target sentences]:

*Yīnwèi liǎng-míng-xuésheng bùzhì(-le) měi-jiān-jiàoshì,*

because two-clf-student decorate(-pfv) every-clf-classroom

*zhè-cì-jiàoshì-bùzhì méiyǒu chūxiàn-le hěn dà de pīlòu.*

this-clf-classroom-decoration not happen-pfv very big link mistake

‘Because two students decorate/decorated every classroom, the decoration task was completed successfully and smoothly this time.’

**(15) ‘if’ type adverbial clause:** *yaoshi_le* condition & *yaoshi_*no *le* condition

[Context]: 振华学校的传统是，每月月末由一个班级负责全校所有教室的布置任务。以往教室布置的任务，都是一个学生负责一间教室，教室布置任务常常也是敷衍了事。很多学生和老师都建议，这次布置教室应该是每间教室都由两名学生负责。两个学生可以分工合作，互相监督，应该不会出现很大的纰漏。

[word-for-word glosses of the written context]:

*Zhènhuá-xuéxiào de chuántǒng shì, měi-yuè-yuèmò yóu*

Zhenhua-school link tradition is every-month-end.of.month by

*yī-ge-bānjí fùzé quán-jiào suǒyǒu jiàoshì de bùzhì*

one-clf-class responsible all-school all classroom link decoration

*rènwu. yǐwǎng jiàoshì bùzhì de rènwu, dōu shì*

task in.the.past classroom decoration link task all foc

*yī-ge-xuésheng fùzé yī-jiān-jiàoshì, jiàoshì bùzhì*

one-clf-student responsible one-clf-classroom classroom decoration

*rènwu chángcháng yě shì fūyǎnliǎoshì.* hěnduō xuésheng

task often also foc perfunctorily many student

*hé lǎoshī dōu jiànyì, zhè-cì-bùzhì-jiàoshì yīnggāi*

and teacher all suggest this-time-decoration-classroom should

*shì měi-jiān-jiàoshì dōu yóu liǎng-míng-xuésheng fùzé.*

be every- clf-classroom all by two- clf-student responsible

*liǎng-gè-xuésheng kěyǐ fēngōng hézuò, hùxiāng jiāndū,*

two- clf-student can share.work collaborate each.other supervise

*yīnggāi bù huì chūxiàn hěn dà de pīlòu.*

should not possible happen very big link mistake

‘At Zhenhua High School, one class is responsible for the decorations of all classrooms at the end of each month. In the past, a class often sent one student to decorate each classroom, and students often did not take the decoration task seriously. Many students and teachers suggest that, for each classroom, there should two students working together. Two students can collaboratively complete the decoration task for a classroom, then the decoration task should be completed successfully and smoothly in the end.’

[Target sentences]: 要是两名学生布置**(了)**每间教室，这次教室布置就不会出现很大的纰漏。

[word-for-word gloss of the target sentences]:

*Yàoshi liǎng-míng-xuésheng bùzhì(-le) měi-jiān-jiàoshì,*

if two-clf-student decorate(-pfv) every-clf-classroom

*zhè-cì-jiàoshì-bùzhì* jiù bù huì *chūxiàn hěn dà de pīlòu.*

this-clf-classroom-decoration then not possible happen very big link mistake

‘If two students decorate/decorated every classroom, the decoration task would be completed successfully and smoothly this time.’

**Set 6**

In this set, the embedded verb is a durative verb: *kānshǒu* ‘guard’, and the existential quantificational phrase is in the form of *sān* ‘three’ + classifier + noun.

**(16) ‘although’ type adverbial clause:** *suiran_le* condition & *suiran_*no *le* condition

[Context]: 以往新华体育馆办大型活动时，发生过多次小偷偷窃钱包、最后小偷逃走的事情。现如今，新华体育馆办大型活动时的安保规则是，每个出口都安排三名警察看守，以检查出入人员。昨天的歌迷会又发生了多起小偷偷窃钱包的事情，虽然每个出口都有三名警察看守，小偷最后还是溜走了。

[word-for-word glosses of the written context]:

*Yǐwǎng xīnhuá-tǐyùguǎn bàn dàxíng-huódòng shí, fāshēng-guò*

In.the.past Xinhua-stadium hold big-event when happen-pfv

*duōcì xiǎotōu tōuqiè qiánbāo, zuìhòu xiǎotōu táozǒu*

many.times thief steal wallet in.the.end thief flee

*de shìqíng. Xiànrújīn, Xīnhuá tǐyùguǎn bàn dàxíng huódòng*

rel matter nowadays Xinhua-stadium hold big-event

*shí de ānquán bǎowèi guīzé shì, měi-gè-chūkǒu*

when link security guard rule is every-clf-exit

*dōu ānpái sān-míng-jǐngchá kānshǒu, yǐ jiǎnchá chūrù-rényuán.*

all assign three-clf-policeman guard in.order.to check gateway-people

*zuótiān de gēmí-huì fāshēng-le duō-qǐ tōuqiè-qiánbāo de*

yesterday link fan.gathering happen-pfv a.few-clf steal-wallet rel

*ànjiàn, suīrán měi-gè-chūkǒu dōu yǒu sān-míng-jǐngchá*

cases although every- clf-exit all have three- clf-policeman

*kānshǒu, xiǎotōu zuìhòu háishi liūzǒu-le*

guard thief in.the.end still slip.away-pfv

‘In the past, when Xinhua Stadium held a public event, there have been many thieves stealing wallets and fleeing at the end. Nowadays, the arrangement for the security forces at Xinhua Stadium is, putting three police guards at each exit, to do the security check. Yesterday, there was a gathering for fan meeting and there were quite a few instances of many thieves stealing wallets. Although there were three policemen at each exit, the thieves still slipped away from the Stadium.’

[Target sentences]: 虽然三名警察看守**(了)**每个出口，小偷还是从活动现场溜走了。

[word-for-word gloss of the target sentences]:

*suīrán sān-míng-jǐngchá kānshǒu(-le) měi-gè-chūkǒu*

although three- clf-policeman guard(-pfv) every- clf-exit

*xiǎotōu háishi cóng huódòng-xiàncháng liūzǒu-le*

thief still from event.site slip.away-pfv

‘Although three police officers guard(ed) each exit, the thieves still slipped away from the Stadium.’

**(17) ‘because’ type adverbial clause:** *yinwei_le* condition & *yinwei_*no *le* condition

[Context]: 以往新华体育馆办大型活动时，发生过多次小偷偷窃钱包、最后小偷逃走的事情。现如今，新华体育馆办大型活动时的安全保卫规则是，每个出口都安排三名警察看守，以检查出入人员。昨天的歌迷会发生了多起偷窃钱包的案件，可是因为每个出口都有三名警察看守，最后小偷无处可逃，都被抓住了。

[word-for-word glosses of the written context]:

*Yǐwǎng xīnhuá-tǐyùguǎn bàn dàxíng-huódòng shí, fāshēng-guò*

In.the.past Xinhua-stadium hold big-event when happen-pfv

*duōcì xiǎotōu tōuqiè qiánbāo, zuìhòu xiǎotōu táozǒu*

many.times thief steal wallet in.the.end thief flee

*de shìqíng. Xiànrújīn, Xīnhuá tǐyùguǎn bàn dàxíng huódòng*

rel matter nowadays Xinhua-stadium hold big-event

*shí de ānquán bǎowèi guīzé shì, měi-gè-chūkǒu*

when link security guard rule is every-clf-exit

*dōu ānpái sān-míng-jǐngchá kānshǒu, yǐ jiǎnchá chūrù-rényuán.*

all assign three-clf-policeman guard in.order.to check gateway-people

*zuótiān de gēmí-huì fāshēng-le duō-qǐ tōuqiè-qiánbāo de*

yesterday link fan.gathering happen-pfv a.few-clf steal-wallet rel

*ànjiàn, kěshì yīnwèi měi-gè-chūkǒu dōu yǒu sān-míng-jǐngchá*

cases but because every- clf-exit all have three- clf-policeman

*kānshǒu, zuìhòu xiǎotōu wúchǔ-kě-táo,*

guard in.the.end thief nowhere-to-hide

*dōu bèi zhuāzhù-le.*

all by arrest-pfv

‘In the past, when Xinhua Stadium held a public event, there have been many thieves stealing wallets and fleeing at the end. Nowadays, the arrangement for the security forces at Xinhua Stadium is, putting three police guards at each exit, to do the security check. Yesterday, there was a gathering for fan meeting and there were quite a few instances of many thieves stealing wallets. Because there were three policemen at each exit, the thieves were not able to slip away from the Stadium and all got caught at the end.’

[Target sentences]: 因为三名警察看守**(了)**每个出口，小偷没能从活动现场溜走。

[word-for-word gloss of the target sentences]:

*yīnwèi sān-míng-jǐngchá kànshǒu(-le) měi-gè-chūkǒu,*

because three-clf-policeman guard(-pfv) every-clf-exit

*xiǎotōu méi néng cóng huódòng-xiàncháng liūzǒu.*

thief not able.to from event.site slip.away

‘Because three police officers guard(ed) each exit, the thieves were not able to slip away from the Stadium.’

**(18) ‘if’ type adverbial clause:** *yaoshi_le* condition & *yaoshi_*no *le* condition

[Context]: 以往新华体育馆办大型活动时，每个出口只有一名警察看守。由于安保力量不够，以前发生过多次小偷偷窃钱包、最后小偷逃走的事情。最近的新华体育馆安保讨论会上，大家跟片区派出所负责人老李建议：加强安保力量，每个出口都安排三名警察把守，检查出入人员。这样的话，即使发生小偷偷窃东西的事情，小偷也不可能从活动现场溜走。

[word-for-word glosses of the written context]:

*Yǐwǎng xīnhuá-tǐyùguǎn bàn dàxíng-huódòng shí, měi-gè-chūkǒu*

In.the.past Xinhua-stadium hold big-event when every-clf-exit

*zhīyǒu yī-míng-jǐngchá kànshǒu. yóuyú ānbǎo-lìliáng bù*

only.have one-clf-policeman guard due.to security.force not

*gòu, yǐqián fāshēng-guò duōcì xiǎotōu tōuqiè*

sufficient previously happen-pfv many.times thief steal

*qiánbāo, zuìhòu xiǎotōu táozǒu de shìqíng. Zuìjìn de*

wallet in.the.end thief flee rel matter recent link

*xīnhuá-tǐyùguǎn ānbǎo tǎolùnhuì shàng, dàjiā gēn ānbǎo-fùzérén*

Xinhua-stadium security meeting at staff to security-manager

*lǎolǐ jiànyì: jiāqiáng ānbǎo-lìliáng, měi-gè-chūkǒu dōu ānpái*

LaoLi suggest strengthen security-force every-clf-exit all assign

*sān-míng-jǐngchá bǎshǒu, jiǎnchá chūrù-rényuán. zhèyàng dehuà,*

three-clf-policeman guard check gateway-people in.this.way if

*jíshǐ fāshēng xiǎotōu tōuqiè dōngxī de shìqíng,*

even.if happen thief steal things rel matter

*xiǎotōu yě bù kěnéng cóng huódòng-xiàncháng liūzǒu.*

thief still not possible from event.site slip.away

‘In the past, when Xinhua Stadium held a public event, there was only one police guard at each exit. Due to insufficient security, there have been many thieves stealing wallets and fleeing at the end. At a recent meeting about security, the staff suggested to Mr. Li, the security manager, that the security forces should be strengthened by putting three police guards at each exit and policemen can do security check at the gateway. This way, even if a thief stole something, it is unlikely that the thief could easily slip away from the Stadium.’

[Target sentences]: 要是三名警察看守**(了)**每个出口，小偷就不可能从活动现场溜走。

[word-for-word gloss of the target sentences]:

*yàoshì sān-míng-jǐngchá kànshǒu měi-gè-chūkǒu,*

if three-clf-policeman guard every-clf-exit

*xiǎotōu jiù bù kěnéng cóng huódòng-xiàncháng liūzǒu.*

thief then not possible from event.site slip.away

‘If three police officers guard each exit, a thief is unlikely to slip away from the Stadium.’

# Supplementary Data

Supplementary Data file is submitted as an excel file.

# Supplementary Figures

Figures are submitted individually.
